# Two Functional Epithelial Sodium Channel Isoforms Are Present in Rodents despite Pronounced Evolutionary Pseudogenization and Exon Fusion

**DOI:** 10.1093/molbev/msab271

**Published:** 2021-09-07

**Authors:** Sean M Gettings, Stephan Maxeiner, Maria Tzika, Matthew R D Cobain, Irina Ruf, Fritz Benseler, Nils Brose, Gabriela Krasteva-Christ, Greetje Vande Velde, Matthias Schönberger, Mike Althaus

**Affiliations:** 1 School of Natural and Environmental Sciences, Newcastle University, Newcastle upon Tyne, United Kingdom; 2 Biomedical Imaging, Department of Imaging and Pathology, Faculty of Medicine, KU Leuven, Leuven, Belgium; 3 Institute for Anatomy and Cell Biology, Saarland University School of Medicine, Homburg, Germany; 4 Division of Messel Research and Mammalogy, Senckenberg Research Institute and Natural History Museum Frankfurt, Frankfurt am Main, Germany; 5 Department of Molecular Neurobiology, Max Planck Institute of Experimental Medicine, Göttingen, Germany; 6 Institute for Functional Gene Analytics, Department of Natural Sciences, Bonn-Rhein-Sieg University of Applied Sciences, Rheinbach, Germany

**Keywords:** epithelial sodium channel, ENaC, rodent, evolution, delta-subunit, *SCNN1D*, pseudogene, exon fusion

## Abstract

The epithelial sodium channel (ENaC) plays a key role in salt and water homeostasis in tetrapod vertebrates. There are four ENaC subunits (α, β, γ, δ), forming heterotrimeric αβγ- or δβγ-ENaCs. Although the physiology of αβγ-ENaC is well understood, for decades the field has stalled with respect to δβγ-ENaC due to the lack of mammalian model organisms. The *SCNN1D* gene coding for δ-ENaC was previously believed to be absent in rodents, hindering studies using standard laboratory animals. We analyzed all currently available rodent genomes and discovered that *SCNN1D* is present in rodents but was independently lost in five rodent lineages, including the Muridae (mice and rats). The independent loss of *SCNN1D* in rodent lineages may be constrained by phylogeny and taxon-specific adaptation to dry habitats, however habitat aridity does not provide a selection pressure for maintenance of *SCNN1D* across Rodentia. A fusion of two exons coding for a structurally flexible region in the extracellular domain of δ-ENaC appeared in the Hystricognathi (a group that includes guinea pigs). This conserved pattern evolved at least 41 Ma and represents a new autapomorphic feature for this clade. Exon fusion does not impair functionality of guinea pig (*Cavia porcellus*) δβγ-ENaC expressed in *Xenopus* oocytes. Electrophysiological characterization at the whole-cell and single-channel level revealed conserved biophysical features and mechanisms controlling guinea pig αβγ- and δβγ-ENaC function as compared with human orthologs. Guinea pigs therefore represent commercially available mammalian model animals that will help shed light on the physiological function of δ-ENaC.

## Introduction

Water-to-land transition in the Devonian period, a key event in the evolution of tetrapod vertebrates (Daeschler et al. 2006), required significant physiological adaptations, including efficient mechanisms of sodium and water homeostasis which involve complex transport mechanisms in vertebrate kidneys (Kuo and Ehrlich 2012; Rossier et al. 2015). In tetrapod vertebrates, sodium and water balance is controlled by hormones that match dietary sodium and water intake to their excretion rates. Vasopressin controls aquaporin-mediated renal water reabsorption and the renin–angiotensin–aldosterone system (RAAS) fine-tunes renal sodium reabsorption via epithelial sodium channels (ENaCs) (Rossier et al. 2015).

The canonical ENaC found in mammalian renal distal convoluted tubules and the cortical collecting ducts is composed of three homologous subunits (α, β, γ) which assemble into a heterotrimeric, sodium-selective ion channel (Noreng et al. 2018). ENaCs are constitutively active ion channels, but channel activity can be adjusted by a multitude of regulatory mechanisms and stimuli (Kleyman and Eaton 2020). Whereas hormones such as aldosterone control ENaC subunit expression (Rossier et al. 2015), the abundance of ENaCs in the plasma membrane is controlled by a complex intracellular signaling network that regulates trafficking to and removal from the plasma membrane (Baines 2013). Furthermore, ENaC open probability is affected by the extracellular sodium and proton concentration (Kashlan et al. 2015; Wichmann et al. 2019; Kleyman and Eaton 2020), processing by intra- and extracellular proteases (Kleyman and Eaton 2020), and mechanical stimuli (Althaus et al. 2007; Knoepp et al. 2020). The importance of a precise adjustment of ENaC activity is illustrated by ENaC mutations that lead to severe human diseases. Mutations that result in enhanced (αβγ-) ENaC activity cause Liddle syndrome (Shimkets et al. 1994), a hereditary form of hypertension, whereas mutations reducing ENaC activity cause hypotension and severe salt-wasting (pseudohypoaldosteronism type 1) (Chang et al. 1996).

Three genes coding for α-, β-, and γ-ENaC (*SCNN1A*, *SCNN1B*, and *SCNN1G*, respectively) are present in modern cyclostomes, indicating that ENaC evolved early in vertebrates and likely became part of a machinery that controlled sodium homeostasis when vertebrates migrated to freshwater and terrestrial environments (Hanukoglu and Hanukoglu 2016; Wichmann and Althaus 2020). A fourth ENaC subunit (δ), which is homologous to the α-subunit, appears in lobe-finned fishes (sarcopterygians) and is present in all major tetrapod lineages (Wichmann and Althaus 2020).

Functional characterization of human and amphibian ENaC orthologs revealed that the δ-subunit can form heteromeric channels with the β- and γ-subunits (Waldmann et al. 1995; Babini et al. 2003). Interestingly, the presence of the δ-subunit changes the biophysical properties and molecular regulation of the channel. Compared with αβγ-ENaCs, δβγ-ENaCs have an enhanced activity generating larger ion currents in heterologous expression systems (Haerteis et al. 2009; Wichmann et al. 2018). Several regulatory mechanisms controlling ENaC activity, such as the auto-regulatory control by extracellular sodium ions (a processes termed sodium self-inhibition, SSI), the sensitivity to the extracellular pH, channel processing by proteases, or response to mechanical stimuli, differs between δβγ-ENaCs and αβγ-ENaCs (Haerteis et al. 2009; Wichmann et al. 2018, 2019; Knoepp et al. 2020). However, the physiological function of the δ-subunit remains unknown, and it is unclear whether it evolved as an additional level of ENaC regulation in tetrapod vertebrates or resembles an evolutionary relic of an α-subunit-like ancestor (Wichmann and Althaus 2020).

Despite intensive efforts to elucidate the physiological function of δ-ENaC, to the best of our knowledge, no study up to today has reported a direct functional detection of δ-ENaC current signals in vivo. This is due to the lack of appropriate pharmacological tools to discriminate between αβγ- and δβγ-ENaCs, and lack of suitable model organisms (Paudel et al. 2021). Major advances in understanding the physiology and pathophysiology of canonical αβγ-ENaC were made by manipulating the genes encoding these three ENaC-subunits in mice. Unfortunately, the gene encoding the δ-subunit (*SCNN1D*) is believed to be a pseudogene in rodents, thus limiting research using the most common animal models in physiology and biomedicine. Consistently, Paudel et al. (2021) recently highlighted the need for appropriate rodent animal models in order to shed light on the role of δ-ENaC in health and disease. Apart from their important role as animal models in biomedical research, rodents comprise approximately 40 % of all extant mammalian species (Burgin et al. 2018). The order Rodentia is characterized by striking adaptive and evolutionary radiations, resulting in great diversity, for example, in terms of locomotion, diet, geographical distribution, and ecology (Fabre et al. 2012; Cox and Hautier 2015). Rodents are therefore suitable model organisms for diverse research areas.

To study the enigmatic ENaC δ-subunit in rodents, we examined the existence of functional *SCNN1D* genes in the currently sequenced rodent genomes. First, we found that the *SCNN1D* gene it is not generally absent from rodents but was independently lost in different rodent suborders, including mice and rats. Second, we observed the fusion of two exons and incorporation of intron DNA into the δ-ENaC coding sequence in the Hystricomorpha (a group that includes guinea pigs). Third, we demonstrate that this exon fusion affects a structurally flexible region of the ion channel and does not impair functionality of guinea pig (*Cavia porcellus*) ENaCs in heterologous expression systems. Fourth, we provide evidence for conserved regulatory characteristics of guinea pig αβγ- and δβγ-ENaCs as compared with human orthologs and identify new molecular characteristics that indicate a physiological role in sodium homeostasis. Finally, we aimed to identify patterns in the geographical distribution of rodent species that maintained or lost their functional δ-ENaC in order to shed light on the lack of potential selection pressures resulting in pseudogenization of *SCNN1D*.

## Results

### Standardizing *SCNN1* Gene Nomenclature

**Fig. 1.**
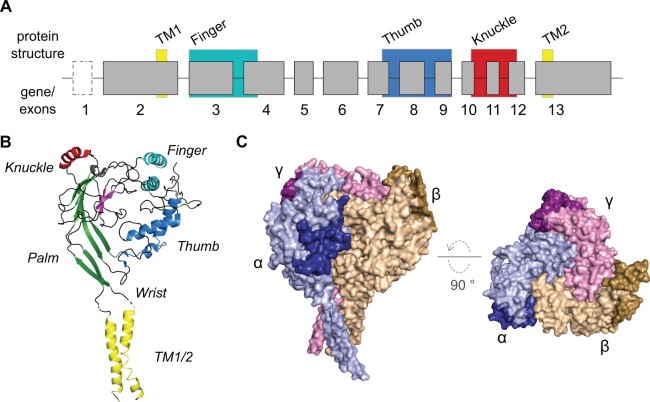
Proposed nomenclature of *SCNN1* genes and structural features of the human αβγ-ENaC. (*A*) The *SCNN1 genes* share a canonical organization in which the coding DNA is distributed over at least 12 exons (exons 2–13). Due to the high variability of exon 2, different predicted start codons located on alternative exons preceding exon 2 as well as the absence of a likely start codon on exon 2 in certain species, make an additional exon(-s) necessary and are therefore depicted in a dashed box. Structural features obtained from the cryo-EM-derived structure of human αβγ-ENaC were imposed to the respective encoding exons. All structural features are highlighted with colored boxes. Only exon sizes and not intron sizes are drawn to scale. (*B*) SCNN1 proteins share an overall hand-like structure, including regions representing “finger,” “thumb,” “palm,” “wrist,” and “knuckle,” holding a “ball of β-sheets” (shown in magenta). Transmembrane regions are termed TM1 and TM2. The image shows the human ENaC α-subunit (Noreng et al. 2018). (*C*) Surface model of the cryo-EM-derived structure of human αβγ-ENaC (Noreng et al. 2018). Gating Relief of Inhibition by Proteolysis (GRIP) domains are highlighted in darker colors.

To categorize the characteristics common to all four *SCNN1* genes and the particular differences that we observed regarding the evolution of *SCNN1D*, and to facilitate future comparisons, we propose a standardization of the nomenclature for the exons of all *SCNN1* genes. Alternative splicing within the “core” coding regions of all *SCNN1* genes, that is, the sequence encompassing both transmembrane regions and, hence, the entire extracellular part, has not yet been reported. Consistent with Saxena et al. (1998), the first transmembrane coding exon should therefore be defined as “exon 2,” and all downstream exons numerated accordingly, concluding with exon 13 (fig. 1*A*). Exon 13 encodes the second transmembrane region and the entire C-terminus. It appears that most but not all translational initiation sites are encoded by exon 2, with some being subject to alternative splicing of upstream exons, for example, human *SCNN1D* which has 16 exons (Giraldez et al. 2012; Wesch et al. 2012; Zhao et al. 2012). To include potential translational start sites encoded by additional and/or alternative start sites upstream of exon 2, we suggest referring to those regions that contain experimentally validated start sites as exon 1. In the case of multiple exons upstream of exon 2, the exon 1 nomenclature should include alphabetical lettering, for example, exon 1a, exon 1b, etc. This standardized nomenclature consistently aligns the coding regions in different exons (gene level) with the structural features (protein level) that have recently been resolved for human αβγ-ENaC (fig. 1*B* and *C*). The extracellular loop of each ENaC subunit resembles a clenched hand holding a “ball-like” structure (fig. 1*B*) (Noreng et al. 2018). The “finger” and “thumb” are considered the major domains involved in ENaC gating, whereas the “palm” and “knuckle” domains contribute to channel regulation via intersubunit interactions (Noreng et al. 2018). This conserved protein structure is also reflected in the organization of the *SCNN1* genes themselves. Exons 2 and 13 encode the transmembrane region, flanking exons 3–12 which encode the extracellular part.

### Distribution of Functional *SCNN1D* in Rodents

**Fig. 2.**
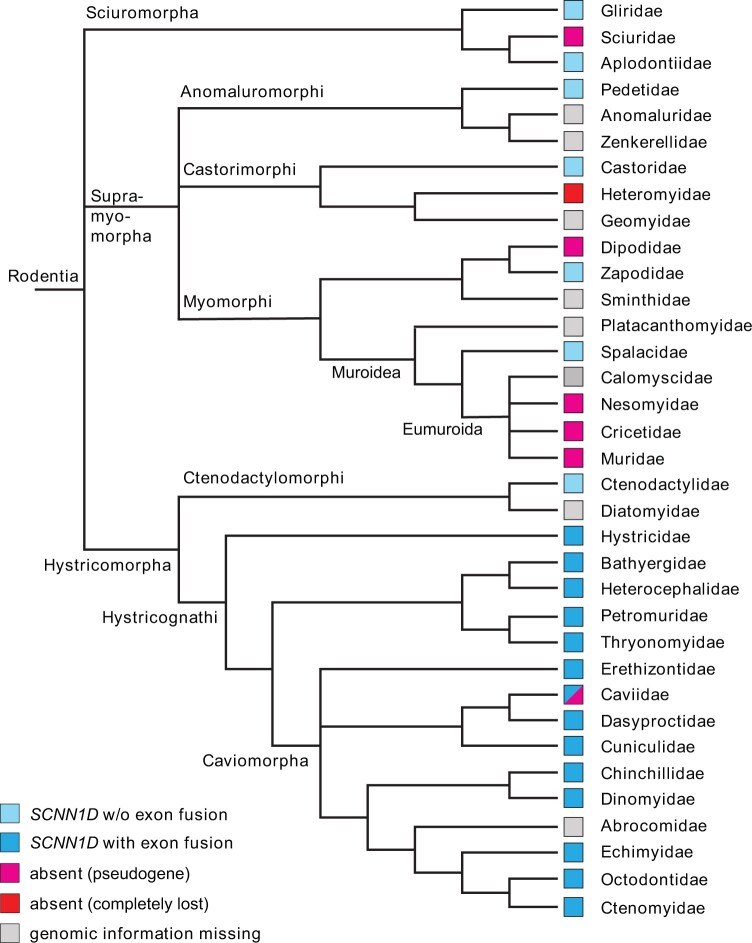
Presence of *SCNN1D* in rodent families. Families marked in magenta include species which lost a functional *SCNN1D*, that is, families with pseudogene versions of *SCNN1D*. *SCNN1D* is completely absent in the Heteromyidae marked in red. All families marked in blue maintained intact *SCNN1D* genes. Families highlighted in dark blue contain species in which exons 11 and 12 of *SCNN1D* are fused to a “super-exon,” whereas light blue families do not include species with *SCNN1D* exon fusion (w/o=without exon fusion). There is currently no available genomic information of species representing the families highlighted in gray. Note that the Caviidae family contains species with and without intact *SCNN1D*. A list of all species that were analyzed is provided in table 1. The classification of rodents into 35 families is based on D’Elía et al. (2019), the taxon Eumuroida was suggested by Steppan et al. (2004).

The absence of *SCNN1D* in the genomic drafts of the rat and mouse genomes prompted us to search for potentially functional *SCNN1D* homologs in rodent genomes of different suborders, with the aim of determining whether *SCNN1D* is generally absent from rodents or specific to a subset thereof. This approach was further motivated by our earlier observation of selective erosion and gene loss within the pseudoautosomal regions of Myomorpha genomes, which is not seen in other suborders (Maxeiner et al. 2020). Currently, 35 rodent families are recognized (D’Elía et al. 2019) (fig. 2), comprising three major clades: Hystricomorpha (a group that includes Old World porcupines, chinchillas, and guinea pigs), Sciuromorpha (squirrels, dormice, and mountain beaver), and Supramyomorpha. The Supramyomorpha are divided into Anomaluromorphi (anomalures and springhares), Castorimorphi (beavers and kangaroo rats), and Myomorphi (mouse-like species) (D’Elía et al. 2019). We investigated the evolutionary fate of the *SCNN1D* gene in all currently available genome sequences within this diverse order (table 1 and supplementary spreadsheet, Supplementary Material online) and made two major observations, a fusion of exons 11 and 12 to a “super-exon” and an independent loss of the *SCNN1D* gene from all rodent suborders (including at least seven families). The generation of a super-exon is exclusive to the suborder Hystricomorpha, specifically to the infraorder Hystricognathi which includes guinea pigs (*C. porcellus*). Within the Sciuromorpha, two of the three families, Aplodontiidae (mountain beaver) and Gliridae (dormouse species), retain a functional *SCNN1D* copy, whereas it is absent from representatives of the Sciuridae family (the squirrel family). Sequence information on members of the Anomaluromorphi is limited but a potentially functional *SCNN1D* gene was found in the South African springhare (*Pedetes capensis*) which belongs to the Pedetidae family. In the Castorimorphi, a full *SCNN1D* reading frame was found in the American beaver (*Castor canadensis*), belonging to the Castoridae, but there was no functional gene in the Gobi jerboa (*Allactata bullata*), illustrating the loss of *SCNN1D* in species within the Dipodidae family. The largest infraorder is the Myomorphi with the superfamilies Dipodoidea and Muroidea. Members of three out of five Muroidea families, the Muridae (rats, mice, gerbils), Cricetidae (hamsters, voles, lemmings, New World rats, and mice), and Nesomyidae (Malagasy rats and mice and specific African species), retained only traces of an evolutionary ancient and, once likely, functional *SCNN1D* gene, which explains failed efforts to clone *SCNN1D* from laboratory mice, rats, or hamsters. Bioinformatic evidence indicates that only the Spalacidae family (blind mole-rats) and the genus *Zapus* within the superfamily Dipodoidea potentially have a functional *SCNN1D* copy, whereas the genera *Jaculus* and *Allactaga* do not. In the Hystricomorpha, multiple stop codons were present in the *SCNN1D* gene of the Patagonian mara (*Dolichotis patagonum*), illustrating gene loss in genera within the Caviidae family, whereas guinea pigs (*C. porcellus*) retained intact open reading frames in *SCNN1D*. Despite the loss of functional *SCNN1D* from several of the families mentioned above, evidence of a decaying *SCNN1D* is still present, embedded between the flanking genes *ACAP3* and *UBE2J2*, which allowed us to integrate sequence information of exon 6 to plot a phylogenetic tree (supplementary fig. 1, supplementary data 2, and supplementary spreadsheet, Supplementary Material online). An exception was the Heteromyidae in which *SCNN1D* is completely absent.

**Table 1.** Presence of an Open Reading Frame (ORF) for *SCNN1D* in Currently Available Rodent Genomes.

	Rodent Family	Species	Genome Assembly	*SCNN1D* ORF	Comments
	Gliridae	*Muscardinus avellanarius*	MusAve_v1_BIUU	Yes	
		*Glis glis*	GliGli_v1_BIUU	Yes	
		*Graphiurus murinus*	GraMur_v1_BIUU	Yes	
	Sciuridae	*Xerus inauris* ^a^	XerIna_v1_BIUU	No	
		*Ictidomys tridecemlineatus*	SpeTri2.0	No	Unsequenced parts of exon 13
		*Cynomys gunnisoni*	ASM1131664v1	No	
		*Spermophilus dauricus*	ASM240643v1	No	
		*Urocitellus parryii*	ASM342692v1	No	
		*Marmota himalayana*	ASM528016v1	No	
		*Marmota marmota*	marMar	No	
		*Marmota monax*	MONAX5	No	
	Aplodontiidae	*Aplodontia rufa*	AplRuf_v1_BIUU	Yes	
	Pedetidae	*Pedetes capensis*	PedCap_v1_BIUU	Yes	Exon 13 information is retrieved from two different sequencing results
	Anomaluridae	No genomes available			
	Zenkerellidae	No genomes available			
	Castoridae	*Castor canadensis*	C.can genome v1.0	Yes	Invasive species
	Heteromyidae	*Dipodomys ordii*	Dord_2.0	No	
	Geomyidae	No genomes available			
	Dipodidae	*Jaculus jaculus*	JacJac1.0	No	
		*Allactaga bullata* ^b^	AllBul_v1_BIUU	No	
	Zapodidae	*Zapus hudsonius*	ZapHud_v1_BIUU	Yes	
	Sminthidae	No genomes available			
	Platacanthomyidae	No genomes available			
	Spalacidae	*Nannospalax galil* ^c^	S.galili_v1.0	Yes	
	Calomyscidae	No genomes available			
	Nesomyidae	*Cricetomys gambianus*	CriGam_v1_BIUU	No	Invasive species
	Cricetidae	*Microtus ochrogaster*	MicOch1.0	No	
		*Cricetulus barabensi griseus* ^d^	CriGri_1.0	No	
		*Peromyscus maniculatus*	Pman_1.0	No	Invasive species
		*Peromyscus leucopus*	UCI_PerLeu_2.1	No	
		*Mesocricetus auratus*	MesAur1.0	No	
	Muridae	*Rattus norvegicus*	Rnor_6.0	No	Invasive species
		*Rattus rattus*	Rrattus_CSIRO_v1	No	Invasive species
		*Grammomys dolichurus surdaster* ^e^	NIH_TR_1.0	No	
		*Mus musculus*	GRCm39	No	Invasive species
		*Mus caroli*	CAROLI_EIJ_v1.1	No	
		*Mastomys coucha*	UCSF_Mcou_1	No	
		*Mus pahari*	PAHARI_EIJ_v1.1	No	
	Ctenodactylidae	*Ctenodactylus gundi*	CteGun_v1_BIUU	Yes	
	Diatomyidae	No genomes available			
	Hystricidae	*Hystrix cristata*	HysCri_v1_BIUU	Yes	
	Bathyergidae	*Fukomys damarensis* ^f^	DMR_v1.0	Yes	Exon 6 yet unsequenced, remaining sequence has normal reading frame
	Heterocephalidae	*Heterocephalus glaber*	HetGla_female_1.0	Yes	
	Petromuridae	*Petromus typicus*	PetTyp_v1_BIUU	Yes	
	Thryonomyidae	*Thryonomys swinderianus*	ThrSwi_v1_BIUU	Yes	
	Erethizontidae	*Erethizon dorsatus*	GSC_porc_1.0	Yes	
	Caviidae	*Dolichotis patagonum*	DolPat_v1_BIUU	No	Internal stop codons
		*Hydrochoerus hydrochaeris*	HydHyd_v1_BIUU	Yes	
		*Cavia porcellus*	Cavpor3.0	Yes	Invasive species
		*Cavia tschudii*	CavTsc_v1_BIUU	Yes	
	Dasyproctidae	*Dasyprocta punctata*	DasPun_v1_BIUU	Yes	
	Cuniculidae	*Cuniculus paca*	CunPac_v1_BIUU	Yes	
	Chinchillidae	*Chinchilla lanigera*	ChiLan1.0	Yes	
	Dinomyidae	*Dinomys branickii*	DinBra_v1_BIUU	Yes	
	Abrocomidae	No genomes available		Yes	
	Echimyidae	*Capromys pilorides*	CapPil_v1_BIUU	Yes	
		*Myocastor coypus*	MyoCoy_v1_BIUU	Yes	Invasive species
	Octodontidae	*Octodon degus*	OctDeg1.0	Yes	
	Ctenomyidae	*Ctenomys sociabilis*	CteSoc_v1_BIUU	Yes	

Note.—Families that include species with intact *SCNN1D* are color coded in blue. Light blue color indicates *SCNN1D* without exon fusion, dark blue color indicates *SCNN1D* with exon fusion. Magenta labels families that include species without functional *SCNN1D* (pseudogene) and red labels families in which *SCNN1D* is completely absent. Families of which genetic information is absent are labeled in gray.

a *Xerus inauris* is assigned to a subgenus, *Geosciurus inauris*.

b *Allactata bullata* is assigned to a subgenus, *Orientallactaga bullata*.

c For analysis of species distribution, the GBIF species *Spalax ehrenbergi* (a now outdated species complex) was used.

d Observation data are also recorded as a separate (now obsolete) species *C. griseus* in GBIF.

e Observation data are also recorded as obsolete synonym *Thamnomys surdaster surdaster* in GBIF.

f *Fukomys damarensis* was previously named *Cryptomys damarensis* as recorded in GBIF (Kock et al. 2006). The species marked as invasive were excluded from the geographical and aridity analyses presented in figure 9.

### Analysis of Four *SCNN1* Genes in the Guinea Pig

**Fig. 3.**
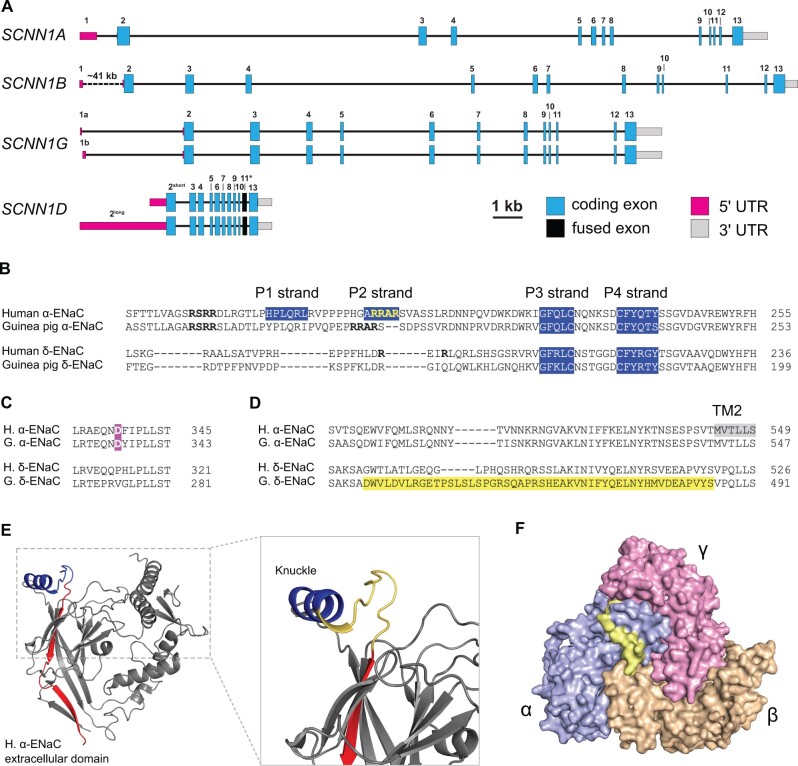
Genomic organization and peptide sequences of guinea pig *SCNN1D*. (*A*) All *SCNN1* family members are depicted relative to scale with the exception of an intron following *SCNN1B* exon 1, which is located roughly 41 kb upstream of *SCNN1B* exon 2. Blue boxes represent coding regions, gray boxes represent 3′-UTR, that is, sequences immediately downstream of the respective stop codons to the first poly-adenylation sequence motif. 5′RACE analyses encompass exon/exon junctions and resulted in 5′-UTR sequence information depicted in magenta. This is reflected in either alternative exons upstream of *SCNN1G* (exons 1a and 1b) or alternative transcriptional start sites of *SCNN1D* formally lacking any upstream exon. The fusion of exon 11 and exon 12 to a “super-exon” 11* is depicted in black. (*B*) Alignment of the amino acid sequences of the extracellular GRIP domains, based on Noreng et al. (2020), of human and guinea pig α- and δ-ENaC. Consensus sites for the protease furin are highlighted in bold letters. P1 to P4 strands of the Gating Relief of Inhibition by Proteolysis (GRIP) domains are highlighted in blue. (*C*) Alignment of amino acid sequences that are part of the extracellular acidic cleft with the putative sodium coordination sites (Asp-338 in human α-ENaC) highlighted in magenta. (*D*) Alignment of amino acid sequences close to the beginning of the second transmembrane domain (TM2) with the “super-exon” 11* of guinea pig δ-ENaC highlighted in yellow. A full sequence alignment is provided in supplementary data 2, Supplementary Material online. (*E*) The region coded by exons 11 (blue) and 12 (red) are highlighted in the structure of the extracellular domain of the human ENaC α-subunit (Noreng et al. 2020). The magnification shows the region between Gln-509 and Ala-522 of the knuckle domain in yellow. This corresponds to the region of incorporated amino acids in guinea pig δ-ENaC due to exon fusion. (*F*) Surface representation of the extracellular domain of human αβγ-ENaC, highlighting the presence of the region between Q509 and A522 in yellow at the protein surface.

Analyses of the complete *SCNN1D* gene sequences of available rodent genomes revealed that guinea pigs retain a functional *SCNN1D* gene (fig. 2). Caviidae are an interesting rodent family in that the *SCNN1D* gene in this family displays a fusion of exons 11 and 12 to a super-exon (including the incorporation of intron sequences) (fig. 3), whereas some family members (e.g., *D. patagonum*) lost a functional *SCNN1D*. We therefore explored whether the open reading frame of the guinea pig (*C. porcellus*) *SCNN1D* is indicative of a functional gene product, that is, an ion channel with the functional characteristics of known ENaC orthologs (Haerteis et al. 2009; Giraldez et al. 2012; Wichmann et al. 2018).

We outlined the genomic structure of the *SCNN1* genes in *C. porcellus* and added experimental data for the presence of “predicted” exons upstream of exon 2 based on our 5′RACE results. Given the reduced size of the entire *SCNN1D* gene and its anticipated high GC content (table 2), we initially needed to validate the primary DNA sequence of the guinea pig *SCNN1D* gene (Gene ID: 100714892) in order to rule out high-throughput sequencing artifacts. Indeed, resequencing revealed a frame-shift in the very C-terminus. The corrected sequence has been deposited on GenBank (MN187539). *SCNN1A*, *SCNN1B*, and *SCNN1G* have a generally similar gene organization (Hanukoglu and Hanukoglu 2016). The 5′RACE experiments revealed the inclusion of a single exon upstream of exon 2 for *SCNN1A* and *SCNN1B*, whereas in the case of *SCNN1G*, two alternative exons are present (1a or 1b, fig. 3*A*). The *SCNN1D* gene displays three major distinguishing features as compared with its homologs: 1) A collapsed gene size and increased GC content (table 2, fig. 3*A*, and supplementary spreadsheet, Supplementary Material online); 2) two alternative transcriptional start sites (short and long versions of exon 2) and the absence of any upstream exons, that is, absence of exon 1; 3) a fusion of exons 11 and 12 forming a “super-exon” (11*) due to the loss of splice donor and acceptor sites (fig. 3*A*).

**Table 2.** Features of Guinea Pig *SCNN1* Genes.

Gene	GC-Content (%)	Size (bases)	GC (cDNA) (%)	GC3 (%)	Amino Acids
*SCNN1A*	50.9	23,116	59.5	80.2	1971
*SCNN1B*	45.2	64,152	56.0	77.4	1926
*SCNN1G*	45.2	26,612	52.1	68.2	1968
*SCNN1D*	58.0	6,438	59.8	76.0	1740

Note.—GC-content reflects percentage of G/C bases in the respective *SCNN1* gene starting from the designated start codon toward the stop codon. GC (cDNA) reflects the overall G/C content in the coding region, GC3 the percentage of G/C in the third position of each codon. Amino acids depict the count of amino acids per *SCNN1* family member.

**Fig. 4.**
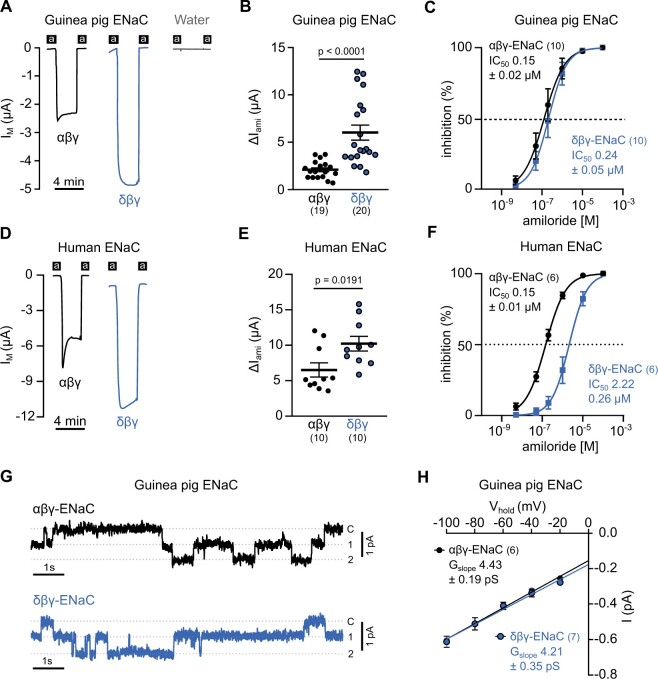
Guinea pig δβγ-ENaC forms a functional channel when expressed in *Xenopus* oocytes. (*A*) Transmembrane current (*I*_M_) traces of oocytes expressing guinea pig αβγ- and δβγ-ENaC as well as water-injected control oocytes at −60 mV holding potential. Application of 100 µM amiloride is represented by black bars (a). (*B*) Amiloride-sensitive current fractions (Δ*I*_ami_) for guinea pig αβγ- and δβγ-ENaCs (Student’s unpaired *t*-test with Welch’s correction). (*C*) Amiloride IC_50_ values were determined from concentration-response experiments for guinea pig αβγ- (black) and δβγ-ENaC (blue). (*D*) Representative *I*_M_ traces of oocytes expressing human αβγ- and δβγ-ENaC at −60 mV holding potential. (*E*) Δ*I*_ami_ for human αβγ- and δβγ-ENaC (Student’s unpaired *t*-test). (*F*) Amiloride IC_50_ values for human αβγ- (black) and δβγ-ENaC (gray) as determined from concentration-response experiments. (*G*) Representative current traces of guinea pig αβγ- and δβγ-ENaC expressing oocytes from cell-attached patch-clamp recordings at a holding potential of −100 mV (c=closed; 1–2, number of open channels). (*H*) Slope conductance (*G*_slope_) of guinea pig αβγ- and δβγ-ENaC, derived from linear regression of unitary channel conductance at holding potentials between −100 to −20 mV. Numbers in parentheses indicate (*n*).

The amino acid sequence of guinea pig δ-ENaC was analyzed in comparison with human δ-ENaC and the α-subunits of both species (fig. 3*B* and supplementary data 2, Supplementary Material online). Four major differences in key regulatory motifs were observed between the δ- and α-subunits: 1) A domain that is unique to ENaC, and referred to as the Gating Relief of Inhibition by Proteolysis (GRIP) domain (fig. 1*C*) (Noreng et al. 2018), is shorter in the δ-subunits, particularly in the regions that correspond to the P1 and P2 strands of the α-subunits (Noreng et al. 2020) (fig. 3*B*); 2) consensus sites for the protease furin, which flank the P1 strand of the GRIP domain, are partly (for human δ-ENaC) or completely (for guinea pig δ-ENaC) absent (fig. 3*C*); 3) a key residue that is involved in the coordination of sodium ions and sodium self-inhibition (Asp-338 in human α-ENaC; Noreng et al. 2020) is present in guinea pig α-ENaC (Asp-336), but absent in both human and guinea pig δ-ENaC (fig. 3*C*); 4) in comparison with human δ-ENaC, guinea pig δ-ENaC has a slightly longer “knuckle” region due to the fusion of exons 11 and 12 to a super-exon, adding five amino acids (fig. 3*D*). According to the extracellular domain of human α-ENaC (Noreng et al. 2020), the additional amino acids are likely incorporated into a region that is structurally flexible and located at the protein surface (fig. 3*E* and *F*).

### General Properties of Guinea Pig αβγ-ENaC and δβγ-ENaC in Comparison to Human ENaC Isoforms

To investigate the functional properties of guinea pig ENaC isoforms and to compare them to known characteristics of human ENaC orthologs, we heterologously expressed guinea pig or human αβγ- or δβγ-ENaC in *Xenopus laevis* oocytes and recorded whole-cell transmembrane currents (*I*_M_) from oocytes clamped at −60 mV using the two-electrode voltage-clamp technique. ENaC activity was determined as fractions of *I*_M_ that were inhibited by 100 µM amiloride, a general ENaC blocker (Δ*I*_ami_). Figure 4*A* shows representative current traces for oocytes expressing either guinea pig αβγ- or δβγ-ENaCs and demonstrates that both guinea pig ENaC-isoforms are functional ion channels. Water-injected control oocytes did not generate any amiloride-sensitive currents. Guinea pig δβγ-ENaC generated significantly larger Δ*I*_ami_ (−6.03 ± 0.79 µA, *n* = 20) than αβγ-ENaC (−2.10 ± 0.21 µA, *n* = 19, *P* < 0.0001, Student’s unpaired *t*-test with Welch’s correction, fig. 4*B*). The half-maximal inhibitory concentration (IC_50_) of amiloride for guinea pig αβγ-ENaC was 0.15 ± 0.02 µM (*n* = 10), not significantly different from the amiloride IC_50_ for δβγ-ENaC (0.24 ± 0.05 µM, *n* = 10, *P* = 0.1095, Mann–Whitney *U* test, fig. 4*C*). As with the guinea pig ENaCs, human δβγ-ENaC generated significantly larger Δ*I*_ami_ (−10.23 ± 3.25 µA, *n* = 10) than human αβγ-ENaC (−6.518 ± 3.192 µA, *n* = 10, *P* = 0.0191, Student’s unpaired *t*-test, fig. 4*D* and *E*). The amiloride IC_50_ values for human ENaCs are consistent with published data (Waldmann et al. 1995) in that human δβγ-ENaC (IC_50_ = 2.22 ± 0.26 µM, *n* = 6) is less sensitive to amiloride than human αβγ-ENaC (IC_50_ = 0.15 ± 0.01 µM, *n* = 6, *P* = 0.0022, Mann–Whitney *U* test, fig. 4*F*). In contrast to αβγ-ENaC, human δβγ-ENaC is more permeable to Na^+^ than Li^+^ (Waldmann et al. 1995). This trait is not shared by guinea pig δβγ-ENaC which is slightly more permeable to Li^+^ over Na^+^ (1.07 ± 0.04, *n* = 19) although not by the same magnitude as guinea pig αβγ-ENaC (1.77 ± 0.11, *n* = 13, *P* < 0.0001, Mann–Whitney *U* test).

The single channel conductance of human δβγ-ENaC is 12 pS (Waldmann et al. 1995; Wesch et al. 2012), which is more than twice as large as αβγ-ENaC (4.9 pS; Fronius et al. 2010). This, together with an increased open probability (Haerteis et al. 2009) explains why the Δ*I*_ami_ of oocytes expressing human δβγ-ENaC are larger than of those expressing αβγ-ENaC. We determined the single-channel conductance of guinea pig αβγ- and δβγ-ENaC (fig. 4*G* and *H*). The slope conductances (*G*_slope_, fig. 4*H*) were calculated from linear regressions of recorded unitary conductances at membrane potentials clamped between −100 and −20 mV. Interestingly, the *G*_slope_ of guinea pig αβγ-ENaC (4.43 ± 0.19 pS, *n* = 6) was not significantly different from the *G*_slope_ of guinea pig δβγ-ENaC (4.21 ± 0.35 pS, *n* = 7, *P* = 0.57, unpaired Student’s *t*-test).

### Isoform-Specific Control of ENaC Activity by Proteases and Sodium

**Fig. 5.**
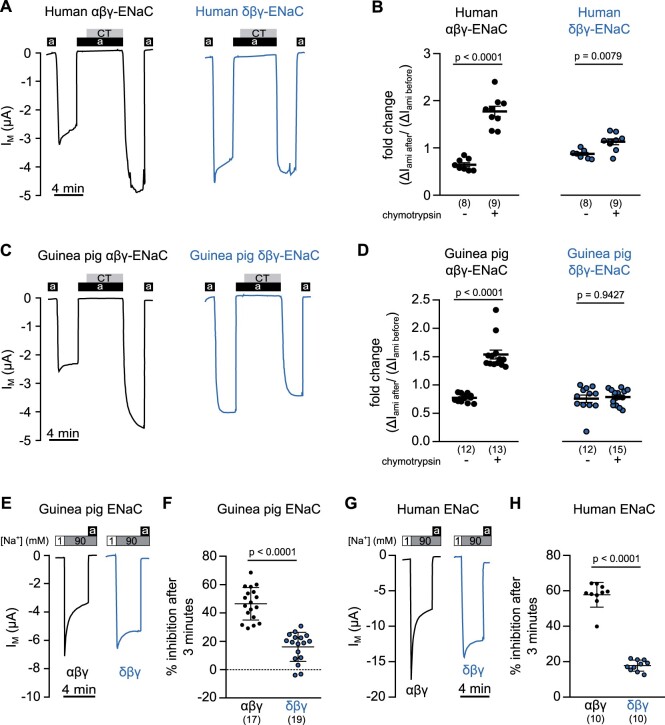
Isoform-specific control of ENaC activity by proteases and sodium. (*A*) Representative transmembrane current (*I*_M_) traces of human αβγ- and δβγ-ENaC expressing oocytes, showing the determination of amiloride-sensitive fractions of *I*_M_ (Δ*I*_ami_) before and after application of chymotrypsin (2 µg/ml, CT, gray bar) with amiloride (100 µM, a, black bar). (*B*) Δ*I*_ami_ were calculated as the difference between *I*_M_ at 3 min after wash-out of amiloride and the *I*_M_ under subsequent presence of amiloride. The fold change (Δ*I*_ami_ after chymotrypsin/Δ*I*_ami_ before chymotrypsin) is shown for human αβγ- and δβγ-ENaC expressing oocytes that were exposed to chymotrypsin as shown in panel (*A*), in comparison with identical control experiments without chymotrypsin (Student’s paired *t*-test). (*C*, *D*) Data from experiments with guinea pig ENaC expressing oocytes that were identical to those shown in panels (*A*) and (*B*). Statistical analysis shown in panel (*D*) was performed with a Mann–Whitney *U* test. (*E*) Representative *I*_M_ trace showing sodium self-inhibition of guinea pig αβγ- and δβγ-ENaC expressing oocytes. Application of amiloride is represented by black bars (a) and [Na^+^] is represented by white (90 mM) and gray (1 mM) bars. The perfusion was at a fast speed of 12 ml/min. (*F*) The percentage of SSI is shown for guinea pig αβγ- and δβγ-ENaC (Student’s unpaired *t*-test). SSI was calculated as (Δ*I*_M_ peak−Δ*I*_M_ 3 min)/Δ*I*_M_ peak×100, where Δ*I*_M_ peak= *I*_M_ under 90 mM [Na^+^] peak − *I*_M_ under 1 mM [Na^+^], and Δ*I*_M_ 3 min=*I*_M_ after 3 min under 90 mM [Na^+^]−*I*_M_ under 1 mM [Na^+^]. (*G*, *H*) Data obtained from experiments using human αβγ- and δβγ-ENaC expressing oocytes. Experiments were identical to those shown in panels (*E*) and (*F*). Statistical analysis of data shown in panel (*F*) was performed using a Student’s unpaired *t*-test with Welch’s correction. Numbers in parentheses indicate (*n*).

Proteolytic processing of ENaC subunits plays a major role in regulating channel activity. Before αβγ-ENaC reaches the plasma membrane, the α- and γ-ENaC subunits are cleaved in the trans-Golgi network by the endoprotease furin (Kleyman and Eaton 2020). The γ-ENaC subunit is cleaved once, whereas the α-ENaC subunit is cleaved twice, thereby removing an inhibitory peptide within the extracellular domain (Kleyman and Eaton 2020). The release of this inhibitory peptide increases ENaC open probability to a moderate level. When furin-processed ENaC reaches the plasma membrane, the γ-ENaC subunit can be additionally cleaved by extracellular proteases (such as prostasin), thereby releasing the inhibitory peptide from the γ-ENaC subunit and further increasing ENaC open probability (Kleyman and Eaton 2020). A well-established protocol for the assessment of proteolytic ENaC activation in *Xenopus* oocytes is the recording of Δ*I*_ami_ before and after exposure to the protease chymotrypsin (Haerteis et al. 2009; Wichmann et al. 2018) (fig. 5). Oocytes expressing guinea pig or human ENaCs were perfused with amiloride. Amiloride was removed for 3 min in order to determine baseline Δ*I*_ami_. Afterward, oocytes were perfused for 5 min with chymotrypsin (2 µg/ml) in the presence of amiloride. Drugs and protease were subsequently removed and Δ*I*_ami_ was determined again (fig. 5*A*). The ratio between the two Δ*I*_ami_ was calculated to reveal fold-changes in ENaC activity due to application of chymotrypsin (fig. 5*B*). To account for changes in ENaC activity over time, identical recordings were performed without chymotrypsin as controls. Consistent with published data (Haerteis et al. 2009), both human ENaC isoforms were activated by the application of extracellular protease. The ratio of the two Δ*I*_ami_ of human αβγ-ENaC expressing oocytes was 0.65 ± 0.04 (*n* = 8) under control conditions, and significantly increased to 1.77 ± 0.11 (*n* = 9; *P* < 0.0001, Student’s paired *t*-test) after chymotrypsin exposure (fig. 5*A* and *B*). For oocytes expressing human δβγ-ENaC, the ratio of the two Δ*I*_ami_ was 0.87 ± 0.03 (*n* = 8) in protease-free controls and increased significantly to 1.13 ± 0.06 (*n* = 9, *P* = 0.0079, Student’s paired *t*-test, fig. 5*A* and *B*) in the presence of chymotrypsin. Chymotrypsin thus leads to a much stronger activation of human αβγ-ENaC than human δβγ-ENaC. Similarly, in oocytes expressing guinea pig αβγ-ENaC, the ratio of the two Δ*I*_ami_ after exposure to chymotrypsin was 1.52 ± 0.08 (*n* = 13), which was significantly larger than the chymotrypsin-free control (0.76 ± 0.02, *n* = 12, *P* < 0.0001, Mann–Whitney *U* test, fig. 5*C* and *D*). By contrast, oocytes expressing guinea pig δβγ-ENaC did not display any differences in the ratio of the two Δ*I*_ami_ following the application of chymotrypsin (0.78 ± 0.03, *n* = 15) or in the chymotrypsin free control group (0.75 ± 0.06, *n* = 12, *P* = 0.9427, Mann–Whitney *U* test, fig. 5*C* and *D*). In summary, whereas αβγ-ENaC is profoundly activated by chymotrypsin, activity of δβγ-ENaC was less affected by protease treatment in both mammalian ENaC orthologs.

In addition to proteolytic processing of ENaC subunits, extra- and intracellular sodium concentrations are important determinants of ENaC activity. ENaC-mediated transmembrane currents typically reduce over time through either feedback inhibition driven by an increase in intracellular sodium concentration or through SSI driven by an increase in extracellular sodium concentration (Chraïbi and Horisberger 2002). The magnitude of SSI was calculated as the percentage of the ENaC-mediated current that declined within 3 min after rapidly switching the sodium concentration in the (extracellular) perfusion solution from 1 to 90 mM sodium (fig. 5*E*). SSI of guinea pig δβγ-ENaC (16.09 ± 2.49%, *n* = 17) was significantly smaller than SSI of guinea pig αβγ-ENaC (46.52 ± 2.708%, *n* = 18; *P* < 0.0001, Student’s unpaired *t*-test, fig. 5*F*). Under the same experimental conditions, the SSI of the human ENaC isoforms were similar to guinea pig ENaCs in that human δβγ-ENaC showed a significantly smaller SSI (17.72 ± 0.99%, *n* = 10) than human αβγ-ENaC (57.68 ± 2.21%, *n* = 10, *P* < 0.0001, Mann–Whitney *U* test, fig. 5*G* and *H*).

### δβγ-ENaC Activity Is Uncoupled from Extracellular Sodium Concentrations

**Fig. 6.**
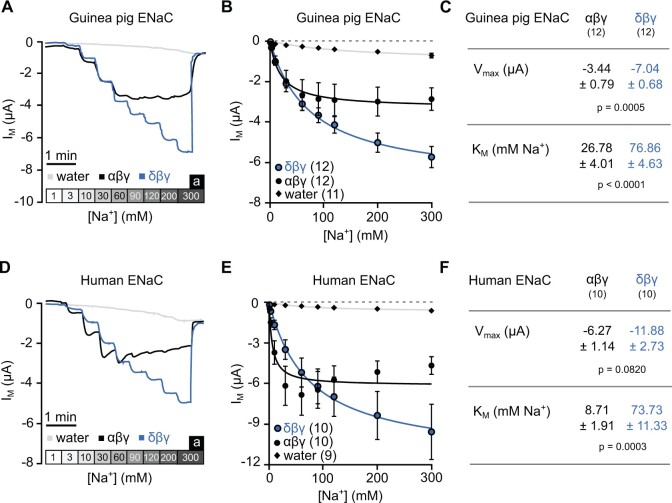
Both human and guinea pig δβγ-ENaC have increased activity compared with αβγ-ENaC at high extracellular Na^+^ concentrations. (*A*) Representative transmembrane current (*I*_M_) traces for guinea pig αβγ- and δβγ-ENaC expressing oocytes as well as water-injected control oocytes. Boxes shaded in gray represent the different extracellular [Na^+^] in mM. (*B*) The *I*_M_ values of guinea pig ENaC expressing oocytes were plotted against the extracellular [Na^+^] and fitted to the Michaelis–Menten equation allowing the estimation of *V*_max_ and the *K*_M_. (*C*) The *V*_max_ values and *K*_M_ values of guinea pig αβγ- and δβγ-ENaC expressing oocytes (Mann–Whitney *U* test). (*D–F*) Similar to A–C except for oocytes expressing human ENaC orthologs and separate water-injected control oocytes. Data shown in panel (*F*) were statistically analyzed with Student’s unpaired *t*-test with Welch’s correction. Numbers in parentheses indicate (*n*).

The control of ENaC activity by SSI avoids excessive uptake of sodium ions into the cells under conditions of high extracellular sodium concentrations (Kleyman et al. 2018). Thus, the reduced SSI in guinea pig and human δβγ-ENaC isoforms indicates uncoupling between ENaC activity and the extracellular sodium concentrations ([Na^+^]). We therefore increased the [Na^+^] gradually from 1 to 300 mM, whereas the *I*_M_ of ENaC expressing oocytes was being recorded. Osmolarity of the extracellular solution was kept constant using N-methyl-d-glucamine as a Na^+^ substitute. Although the *I*_M_ of guinea pig αβγ-ENaC expressing oocytes did not further increase at extracellular [Na^+^] beyond 60 mM, there was a continuous increase in *I*_M_ of guinea pig δβγ-ENaC expressing oocytes across the employed range of extracellular [Na^+^] used (fig. 6*A*). Values of *I*_M_ at different extracellular [Na^+^] were fitted to Michaelis–Menten kinetics (fig. 6*B*) to estimate the maximal current generated by each ENaC isoform (*V*_max_) and the extracellular [Na^+^] at which half the *V*_max_ is achieved (*K*_M_) (fig. 6*C*). Guinea pig δβγ-ENaC has a significantly higher *K*_M_ (76.86 ± 4.63 mM Na^+^, *n* = 12) than guinea pig αβγ-ENaC (26.78 ± 4.01 mM Na^+^, *n* = 12, *P* < 0.0001, Mann–Whitney *U* test), indicating that the presence of the δ-subunit causes increased ENaC activity at high extracellular [Na^+^]. The *V*_max_ of guinea pig δβγ-ENaC was also significantly larger (−7.04 ± 0.68 µA, *n* = 12) than that of αβγ-ENaC (−3.44 ± 0.79 µA, *n* = 12, *P* = 0.0005, Mann–Whitney *U* test), consistent with the larger Δ*I*_ami_ recorded in oocytes expressing δβγ-ENaCs as compared with those expressing αβγ-ENaC (fig. 4*A* and *B*).

Similar results were obtained with human ENaC isoforms (fig. 6*D*–*F*). Human δβγ-ENaC also had a significantly higher *K*_M_ (73.73 ± 11.33 mM Na^+^, *n* = 10) than αβγ-ENaC (8.71 ± 1.91 mM Na^+^, *n* = 10, *P* = 0.0003, Student’s unpaired *t*-test with Welch’s correction). *V*_max_ of human δβγ-ENaC (−11.88 ± 2.73 µA, *n* = 10) was larger than αβγ-ENaC (−6.27 ± 1.14 µA, *n* = 10), but statistical significance was not reached (*P* = 0.082, Student’s unpaired *t*-test with Welch’s correction, fig. 6*F*). Nevertheless, the presence of the δ-subunit appears to increase ENaC activity at high extracellular [Na^+^] in both human and guinea pig ENaCs.

### Reduced Sodium Self-Inhibition Is Pivotal to ENaC Activity at High Extracellular Sodium Concentrations

**Fig. 7.**
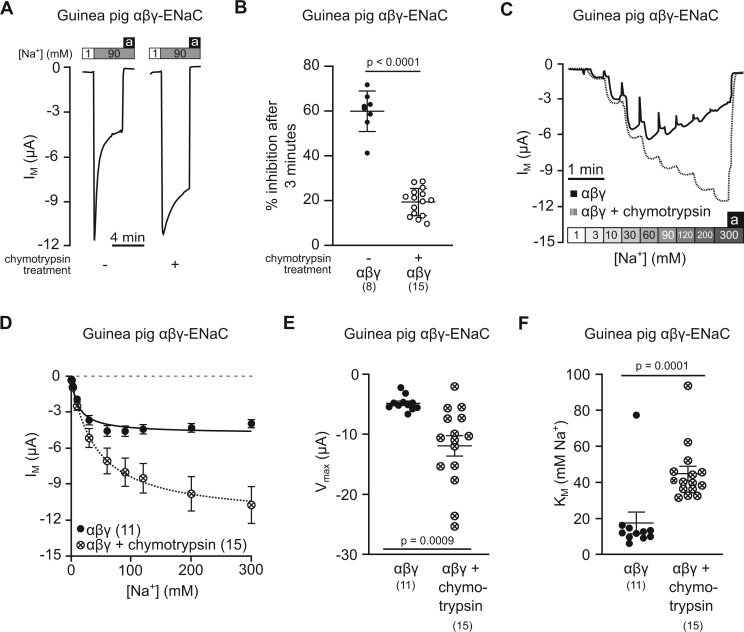
Reduced sodium self-inhibition is pivotal to ENaC activity at high extracellular Na^+^ concentrations. (*A*) Representative transmembrane current (*I*_M_) traces showing sodium self-inhibition (SSI), determined with guinea pig αβγ-ENaC expressing oocytes, with and without prior incubation with chymotrypsin (2 µg/ml in NMDG-ORS for 5 min). Application of amiloride (100 µM) is represented by black bars (a) and [Na^+^] is represented by white (90 mM) and gray (1 mM) bars. The perfusion speed was 12 ml/min. (*B*) The percentage of SSI was plotted for guinea pig αβγ-ENaC with and without prior incubation with chymotrypsin (Student’s unpaired *t*-test). SSI was calculated as (Δ*I*_M_ peak−Δ*I*_M_ 3 min)/Δ*I*_M_ peak×100, where Δ*I*_M_ peak=*I*_M_ under 90 mM [Na^+^] peak −*I*_M_ under 1 mM [Na^+^], and Δ*I*_M_ 3 min=*I*_M_ after 3 min under 90 mM [Na^+^]−*I*_M_ under 1 mM [Na^+^]. (*C*) Representative *I*_M_ traces for guinea pig αβγ-ENaC expressing oocytes with and without prior incubation with chymotrypsin (2 µg/ml for 5 min) across a range of extracellular Na^+^ concentrations ([Na^+^]), gray-shaded boxes). (*D*) The *I*_M_ from experiments shown in panel (*C*) were plotted against the extracellular [Na^+^] and fitted to the Michaelis–Menten equation allowing the estimation of the maximum *I*_M_ (*V*_max_) and the [Na^+^] at which half of *V*_max_ is reached (*K*_M_). (*E*) The *V*_max_ values of guinea pig αβγ-ENaC with and without prior incubation with chymotrypsin (Student’s unpaired *t*-test with Welch’s correction). (*F*) The *K*_M_ values of guinea pig αβγ-ENaC with and without prior incubation with chymotrypsin (Mann–Whitney *U* test). Numbers in parentheses indicate (*n*).

Proteolytic processing of ENaC subunits and SSI are linked regulatory processes. Cleavage of human αβγ-ENaC expressed in *Xenopus* oocytes causes a reduction in the magnitude of SSI (Chraïbi and Horisberger 2002) whereas mutations in furin cleavage sites that prevent proteolytic processing in mouse α-ENaC causes increased SSI (Sheng et al. 2006). We recorded SSI of guinea pig αβγ-ENaC with and without prior incubation in chymotrypsin (2 µg/ml in NMDG-ORS) for 5 min (fig. 7*A*). After incubation in chymotrypsin, SSI of guinea pig αβγ-ENaC was 19.28 ± 1.57% (*n* = 15), significantly smaller than the SSI of guinea pig αβγ-ENaCs that were not treated with chymotrypsin (59.83 ± 3.19%, *n* = 8, *P* < 0.0001, unpaired Student’s *t*-test, fig. 7*B*). The *I*_M_ of guinea pig αβγ-ENaCs with and without prior incubation with chymotrypsin were then recorded as the extracellular [Na^+^] was increased from 1 mM to 300 mM (fig. 7*C*). Exposure of guinea pig αβγ-ENaC to chymotrypsin significantly increased *V*_max_ (−11.93 ± 1.69 µA) and *K*_M_ (44.87 ± 4.06 mM Na^+^, *n* = 15) as compared with untreated αβγ-ENaCs (*V*_max_: −4.65 ± 0.37 µA, *n* = 11, *P* = 0.0009, unpaired Student’s *t*-test with Welch’s correction; *K*_M_: 17.56 ± 6.03 mM Na^+^, *P* < 0.0001, Mann–Whitney *U* test, fig. 7*E* and *F*). These data indicate that proteolytic reduction of SSI in guinea pig αβγ-ENaC enhances ENaC activity across the range of extracellular [Na^+^] used and resembles the activity of guinea pig δβγ-ENaC (fig. 6) which has an inherently reduced SSI and lacks proteolytic activation (fig. 5).

### The Reduced Sodium Self-Inhibition of Human and Guinea Pig δβγ-ENaC Generates Increased Activity at High Extracellular Sodium Concentrations

**Fig. 8.**
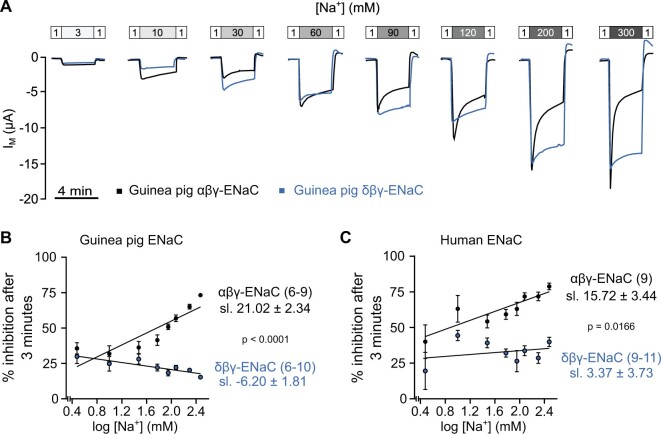
The reduced sodium self-inhibition of human and guinea pig δβγ-ENaC generates increased activity at high extracellular Na^+^ concentrations. (*A*) Representative transmembrane current (*I*_M_) traces of guinea pig αβγ- and δβγ-ENaC at different extracellular Na^+^ concentrations ([Na^+^]). Recordings of a given extracellular [Na^+^] were performed in individual oocytes. The perfusion speed was 16 ml/min. (*B*, *C*) The percentage of sodium self-inhibition for guinea pig and human ENaC expressing oocytes plotted against logarithmic transformations of concentrations of extracellular [Na^+^]. SSI was calculated as (Δ*I*_M_ peak−Δ*I*_M_ 3 min)/Δ*I*_M_ peak×100, where Δ*I*_M_ peak=*I*_M_ under X mM [Na^+^] peak −initial *I*_M_ under 1 mM [Na^+^], and Δ*I*_M_ 3 min=*I*_M_ after 3 min under X mM [Na^+^]−initial *I*_M_ under 1 mM [Na^+^]. Slopes (sl.) were derived from linear regressions and *P* values, derived from ANCOVA, demonstrate the difference between slopes. Numbers in parentheses indicate (*n*).

We observed that both human and guinea pig δβγ-ENaC are more active at high extracellular [Na^+^] than αβγ-ENaCs (fig. 6). Human and guinea pig δβγ-ENaC have a reduced SSI (fig. 5) and the *I*_M_–[Na^+^] relationship is altered by a change in the magnitude of SSI (fig. 7). We therefore hypothesized that the magnitude of SSI of guinea pig and human δβγ-ENaC is reduced across the employed range of extracellular [Na^+^], thereby establishing increased ENaC activity. We recorded SSI of guinea pig αβγ- and δβγ-ENaC expressing oocytes at extracellular [Na^+^] between 3 and 300 mM (fig. 8*A*). The magnitude of SSI in guinea pig αβγ-ENaC (fig. 8*B*) increased steadily from 35.69 ± 3.98% at 3 mM extracellular [Na^+^] to 73.15 ± 1.25% at 300 mM extracellular [Na^+^], whereas the magnitude of SSI of guinea pig δβγ-ENaC appeared to decline slightly as extracellular [Na^+^] increased. The relationship between SSI and the extracellular [Na^+^] was described by the slope derived from linear regression of SSI at logarithmic transformations of extracellular [Na^+^] between 3 and 300 mM (fig. 8*B*). The slope of the regression line of guinea pig αβγ-ENaC (21.02 ± 2.37, *n* = 6–9) was significantly larger than that of δβγ-ENaC (−6.20 ± 1.81, *n* = 6–10, ANCOVA, *P* < 0.0001, *F*_1, 135_ = 84.01). A similar result was obtained when the magnitudes of SSI of human ENaCs orthologs were estimated using the same protocol (fig. 8*C*). The slope of the linear regression of human αβγ-ENaC (15.72 ± 3.44, *n* = 9) was also significantly larger than that of δβγ-ENaC (3.37 ± 3.73, *n* = 9–11, ANCOVA, *P* = 0.0166, *F*_1, 142_ = 5.879, fig. 8*C*). In sum, these data indicate that the reduced SSI uncouples the control of δβγ-ENaC activity from extracellular [Na^+^], thereby leading to enhanced ENaC-mediated sodium uptake across a wide range of extracellular sodium concentrations.

### Functional Gene Coding for δ-ENaC Is not Associated with Habitat Aridity across Rodentia

**Fig. 9.**
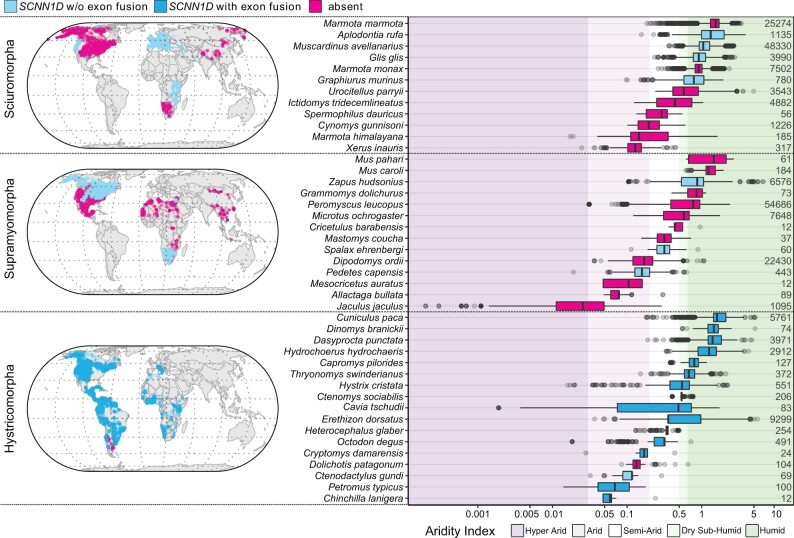
The loss of *SCNN1D* does not generally correlate with habitat aridity. Geolocation data of the rodent species listed in table 1 were extracted from the Global Biodiversity Information Facility (GBIF) and were used to plot the global distribution of noninvasive rodent species of the clades Sciuromorpha, Supramyomorpha, and Hystricomorpha. Global distribution of individual species is provided in supplementary figures 2–5, Supplementary Material online. Observations for the indicated species were also plotted with corresponding habitat aridity. Mixed effects models suggest that the absence or presence of functional *SCNN1D* in the investigated species does not explain any significant proportion variation in habitat aridity. The colors indicate absence or presence of functional *SCNN1D*: Light blue=*SCNN1D* without exon fusion; Dark blue=*SCNN1D* with exon fusion; Magenta=species without functional *SCNN1D* (pseudogene and complete gene loss). Background colors differentiate habitats following the generalized climate classification scheme for aridity index values (Middleton and Thomas 1997). Species are ordered based on ascending median aridity within each clade. The numbers to the right of the plots indicate the number of GBIF observations that were extracted for each species.

In mammals, ENaC plays an important role in fine-tuning renal sodium reabsorption and extracellular fluid volume as part of the RAAS (Schild 2010). It is therefore considered a key player in the control of sodium and water homeostasis. Interestingly, mammals living in dry (arid) habitats appear to have higher basal levels of the RAAS (Donald and Pannabecker 2015), and recent studies indicated that enhanced ENaC activity drives renal water reabsorption and fecal dehydration in desert mammals (Wu et al. 2014; Zhang et al. 2019). Since δβγ-ENaCs have an enhanced activity as compared with αβγ-ENaCs and would therefore enhance sodium and, consequently, water conservation, we explored whether the presence of the δ-subunit in a rodent species might correlate with associated environmental factors. Geolocation data extracted from the Global Biodiversity Information Facility (GBIF) were used to plot the global distribution of noninvasive rodent species that maintained a functional *SCNN1D* and those that lost it (fig. 9 and supplementary figs. 2–5, Supplementary Material online). Invasive rodent species, as described in the Global Invasive Species database, were excluded from the analysis in order to focus on the natural species’ distributions (supplementary fig. 5, Supplementary Material online). In Sciuromorpha, loss of *SCNN1D* was observed in North American, East Asian, and South African species (fig. 9). Interestingly, most of the species lacking *SCNN1D* are adapted to cold and warm arid environments (supplementary fig. 2, Supplementary Material online) (Wilson et al. 2017). In Supramyomorpha, species lacking *SCNN1D* are widely distributed across various climates in North America, Africa, and South-East Asia (fig. 9 and supplementary fig. 3, Supplementary Material online). Interestingly, all Hystricomorpha species we investigated maintained functional *SCNN1D* independent of habitat aridity, except for the Patagonian mara (*D. patagonum*) (fig. 9 and supplementary fig. 4, Supplementary Material online). Based on a potential habitat-dependent distribution of rodents with and without functional *SCNN1D* among Sciuromorpha, we tested whether its presence partially explained variation in habitat aridity. Species with and without the gene (*n* = 43) were found distributed across a wide range of habitat aridities (fig. 9). However, inclusion of gene presence did not significantly improve model fit compared with the null model of random species effects only (χ^2^_(1)_ = 1.20, *P* = 0.273), with random species effects alone explaining 49.5% of the observed variance in aridity (cf. 52.2% conditional *r*^2^ for the model with gene presence). Modeling more complex phylogeny (species nested within clade) could not be resolved due to singular fits of the mixed effects models. Therefore, evidence based on this statistical analysis suggests that the presence of a functional *SCNN1D* does not appear to be associated with observed aridity across Rodentia given species level differences in habitat.

## Discussion

This study closes an intriguing gap in knowledge regarding functional δ-ENaC in rodents, which has puzzled the field of ENaC research for more than 20 years—to the point that rodents had been assumed to lack a functional *SCNN1D* gene (Kleyman and Eaton 2020; Paudel et al. 2021). Based on an investigation of likely functional δ-ENaC-encoding *SCNN1D* genes in rodent genomes, we report that *SCNN1D* is not generally absent from rodents but was independently lost in at least five rodent clades, including the Muridae family (rats and mice). We note that previous studies employing RT–PCR indicated the presence of *SCNN1D* in mouse (Nie et al. 2009), but the entire gene has never been fully identified and its gene product has never been cloned. Given the current sequence coverage of the mouse genome, its position would have been identified. Hence, previously reported RT–PCR data (Nie et al. 2009) are likely due to residual *SCNN1D* promoter activity that does not result in a functional mRNA.

The “loss of the *SCNN1D*” gene was actually a loss of function due to frame-shifts, preliminary stop signals or disruptions of splice-donor and splice-acceptor sites, rather than complex genomic rearrangements resulting in a deletion of genomic information, leaving its flanking genes, *UBE2J2* and *ACAP3*, intact. Further, changes to a likely functional *SCNN1D* gene arose in the infraorder Hystricognathi, resulting in a fused super-exon consisting of exons 11 and 12 with the separating intron. Exons 11 and 12 code for the ENaC “knuckle” domain which interacts with the finger domains of the neighboring ENaC subunits (Noreng et al. 2018) and plays an important role in SSI (Chen et al. 2015). Strikingly, the incorporation of additional amino acids into this region of guinea pig δ-ENaC due to the exon fusion does not affect channel functionality. This is likely due to the peripheral location of the knuckle domain at the channel surface, which allows substantial flexibility in contrast to other domains which are, for example, involved in key parts of the gating machinery of the ion channel.

The most parsimonious explanation is that the “super-exon” is a new autapomorphic feature of Hystricognathi. This clade comprises Hystricidae (Old World porcupines), Phiomorpha (African cane rats, dassie, and mole rats), and Caviomorpha (New World hystricognaths. i.e., cavies and allies) (Patterson and Upham 2014). The oldest known Hystricognathi are the caviomorphs *Canaanimys maguiensis*, *Cachiyacuy kummeli*, and *Cachiyacuy contamanensis* from the late Middle Eocene (∼41 Ma) of Peru (Antoine et al. 2012). However, the origin of Hystricognathi is certainly in Asia and dates back into the Early Eocene or even Late Palaeocene (up to 56 Ma), although the oldest phiomorph fossils are known from younger deposits in the Middle Eocene of North Africa (Antoine et al. 2012; Marivaux et al. 2014; Patterson and Upham 2014). The oldest Hystricidae are members of the genus *Atherurus* which were found in Late and Middle Miocene deposits of Pakistan (∼15 Ma) and Egypt (∼11 Ma) (Weers 2005; Mein and Pickford 2006). Thus, we can confidently conclude that the fusion of exons 11 and 12 evolved in the Eocene at the latest because it was already present in the last common ancestor of Hystricognathi. Although the super-exon may not very substantially affect the function of δβγ-ENaC in general, it has been a stable feature for more than 41 Ma.

Functional analyses of guinea pig αβγ- and δβγ-ENaCs expressed in *Xenopus* oocytes allowed us to compare their biophysical and regulatory properties with human ENaC orthologs. Compared with αβγ-ENaCs, we found that guinea pig δβγ-ENaCs: 1) generate larger transmembrane currents; 2) have a reduced sensitivity to extracellular proteases; 3) have a reduced SSI; and 4) exhibit uncoupling between the control of channel activity and the extracellular [Na^+^]. These characteristics are similar to those of the human ENaC orthologs. As a constitutively active ion channel, the transmembrane current generated by ENaC activity depends on the number of active channels in the plasma membrane, the single channel conductance, and the open probability. Previous studies showed that δ-subunit incorporation does not lead to an increased membrane abundance of human (Haerteis et al. 2009) and *Xenopus laevis* δβγ-ENaCs (Wichmann et al. 2018). Assuming, accordingly, that there is also no difference in the membrane abundance between guinea pig δβγ-ENaC and αβγ-ENaC, the observed differences in transmembrane currents and in coupling between ENaC current and the extracellular [Na^+^], are likely caused by different single channel conductance or open probability.

The single channel sodium conductance of human δβγ-ENaC is more than twice as large as that of αβγ-ENaC (Waldmann et al. 1995; Wesch et al. 2012). This seems to be a novel feature of human ENaCs, as the single channel sodium conductances of guinea pig and *Xenopus laevis* αβγ- and δβγ-ENaCs are similar (Wichmann et al. 2018). Unlike the human isoforms, the difference in transmembrane currents between guinea pig ENaC isoforms is not related to a larger single channel conductance of δβγ-ENaC, so that the difference between transmembrane currents of guinea pig αβγ- and δβγ-ENaCs and the relationship between transmembrane currents and extracellular [Na^+^], are likely due to differential ENaC open probabilities. Indeed, proteolytic processing and sodium self-inhibition, key control mechanisms of ENaC open probability (Kleyman and Eaton 2020), are reduced in guinea pig and human δβγ-ENaCs.

The Cryo-EM-derived structure of human αβγ-ENaC reveals a domain that is unique to ENaC and referred to as the Gating Relief of Inhibition by Proteolysis (GRIP) domain (Noreng et al. 2018). The peptide sequence that forms the GRIP domain resides between the α1 and α2 helices of the “finger” domain and contains inhibitory tracts that are released when ENaC is cleaved by proteases. The intracellular protease furin cleaves ENaC within the Golgi apparatus at the consensus site (Arg-X-X-Arg) (Molloy et al. 1992). The human α-ENaC subunit has two furin consensus sites within the GRIP domain and is cleaved twice by furin. This removes the inhibitory peptide and changes ENaC from a near silent channel to one with an intermediate open probability (Kleyman and Eaton 2020). The γ-ENaC subunit is only cleaved once by furin. The activation of membrane bound ENaC is attributed to a second cleavage of γ-ENaC by extracellular proteases, releasing the inhibitory tract from γ-ENaC. This switches ENaC to a high open probability, increasing its activity (Kleyman and Eaton 2020). Consistent with previous reports (Haerteis et al. 2009), human αβγ- and δβγ-ENaCs are activated by extracellular proteases, but the activity increase of human δβγ-ENaC is smaller than that of αβγ-ENaC. Although the activity of guinea pig αβγ-ENaC is increased, the activity of δβγ-ENaC did not change upon application of extracellular protease. Guinea pig δ-ENaCs lack the two furin consensus sites that are present in the α-subunit (Wichmann et al. 2019; Wichmann and Althaus 2020). Further, we showed previously that the presence of the δ-subunit in *Xenopus laevis* δβγ-ENaCs prevents extracellular protease (chymotrypsin) from cleaving the γ-subunit (Wichmann et al. 2019). It is thus likely that the insensitivity of guinea pig δβγ-ENaC to extracellular protease is caused by the absence of furin cleavage sites in δ-ENaC and prevention of γ-subunit cleavage. We also observed that the peptide sequences of human and guinea pig δ-ENaC GRIP domains are shorter than those of α-ENaC in both species, particularly in the region encompassing the P1 and P2 strands of the α-subunits GRIP domains. Removal of the P1 strand was recently suggested to cause the loss of SSI after proteolytic cleavage (Noreng et al. 2020). Thus, the shorter GRIP domains of human and guinea pig δ-ENaC may explain the greatly reduced SSI in human and guinea pig δβγ-ENaCs.

Previous studies demonstrated that proteolytic processing activates ENaC by relieving SSI (Chraïbi and Horisberger 2002). As shown in this and previous studies (Ji et al. 2006), the magnitude of SSI is lower in human δβγ-ENaCs than in human αβγ-ENaCs. In line with the human ENaC isoforms, the magnitude of SSI in guinea pig δβγ-ENaCs is also reduced as compared αβγ-ENaCs. SSI likely involves the coordination of sodium ions in an extracellular region termed the acidic cleft (Kashlan et al. 2015; Wichmann et al. 2019). A key residue that likely coordinates sodium ions was identified the β6–β7 loop of the acidic cleft of human and mouse α-ENaC (Asp-338 and Asp-365, respectively) (Kashlan et al. 2015; Noreng et al. 2020) and of *Xenopus laevis* δ-ENaC (Asp-296), which, in contrast to mammalian δβγ-ENaCs, shows a strong SSI. This conserved residue is absent from human and guinea pig δ-ENaCs (Wichmann et al. 2019), which might further contribute to the reduced SSI in these variants. In addition to the residues in the acidic cleft, intrasubunit interactions between the “finger” and “knuckle” domains of neighboring ENaC subunits are likely required to translate sodium binding in the acidic cleft to a change in the channel gate (Wichmann et al. 2019). This supports the idea that the shorter GRIP domains of human and guinea pig δ-ENaC may contribute to a reduced SSI by restricting conformational changes in the channels. Further, deletion of the “knuckle” domain in the α-subunit of mouse ENaC results in loss of SSI (Chen et al. 2015). The altered structure of the “knuckle” domain due to exon fusion in guinea pig δ-ENaC might therefore also contribute to structural impairments that reduce SSI.

Consistent with previous reports on human ENaC (Chraïbi and Horisberger 2002), the magnitude of SSI increases with the extracellular [Na^+^] in human and guinea pig αβγ-ENaCs, a phenomenon that was suggested to protect epithelial cells from absorbing excess sodium ions (Kleyman et al. 2018). SSI is a dynamic mechanism for regulating ion channel activity that responds instantaneously to changes in urinary [Na^+^]. This coupling of ENaC activity to the extracellular [Na^+^] is absent from human and guinea pig δβγ-ENaCs, resulting in increased ion channel activity as the extracellular [Na^+^] rose above approximately 60 mM. This enhanced activity under high extracellular [Na^+^] is linked to the reduced SSI in these ENaC isoforms. Previous studies on mouse αβγ-ENaC showed that the magnitude of SSI is reduced in proteolytically processed channels (Sheng et al. 2006). In addition, prevention of proteolytic processing of mouse α-ENaC by mutating the furin consensus sites increases the magnitude of SSI (Sheng et al. 2006). Consistently, exposure of guinea pig αβγ-ENaC to chymotrypsin led to SSI reduction and increased ion channel activity at higher extracellular [Na^+^]. Interestingly the *K*_M_ and *V*_max_ of the cleaved αβγ-ENaC were more than twice as big as those of uncleaved αβγ-ENaC, mirroring the difference between the *K*_M_ and *V*_max_ of the human and guinea pig δβγ- and αβγ-ENaC isoforms. In this instance the proteolytically processed αβγ-ENaC behaves like the protease-insensitive δβγ-ENaC and displays greater activity at higher extracellular [Na^+^].

In summary, guinea pig and human δβγ-ENaCs have an increased activity as compared with αβγ-ENaCs, and channel activity is not curbed at high extracellular [Na^+^] due to a strongly reduced SSI. Assuming that these characteristics are conserved among rodent ENaCs, the question of a physiological context in which such properties might be advantageous arises. Key targets for the RAAS are the distal convoluted tubules and cortical collecting ducts in the mammalian kidney, where aldosterone controls αβγ-ENaC expression to match sodium excretion to dietary sodium intake (Palmer and Schnermann 2015). The RAAS evolved in tetrapod vertebrates as an adaptation to a terrestrial environment, and, to the best of our knowledge, there is no tetrapod vertebrate species that lacks genes coding for αβγ-ENaC. Given the physiological importance of αβγ-ENaC, we searched for evidence indicating that δβγ-ENaCs displaying increased activity (as compared with αβγ-ENaC) might be an advantage for rodent species living in particularly arid environments with higher physiological demands on sodium and water homeostasis.

The loss of *SCNN1D* represents an apomorphic feature that evolved independently in all three major rodent clades. In the Sciuromorpha this trait is restricted to the Sciuridae, which at first glance indicates a phylogenetic signal. However, our sciurid sample comprises only ground squirrels of the subfamily Xerinae, more specifically, of the tribes Xerini and Marmotini (Steppan et al. 2004). Thus, representatives of the third xerine tribe Protoxerini and further species of the other sciurid subfamilies need to be investigated in order to test a phylogenetic signal for the respective taxon level. Although our results clearly show that loss of *SCNN1D* cannot generally be related to aridity of the habitat of the investigated species across Rodentia, there appears to be a climate-related signal within the Sciuridae under study. All the investigated Xerini are adapted to cold and warm arid environments like mountain regions, steppes, prairie, and (semi)deserts (Wilson et al. 2017). One exception is *Marmota monax* which is widely distributed from Alaska and Canada into open lowland environments of the eastern United States, covering a wide range of ecosystems and climates (Wilson et al. 2017) (supplementary fig. 2, Supplementary Material online). According to Polly (2003), *Marmota monax* subspecies evolved and differentiated over several glacial cycles within the last 750,000 years whereas other extant North American *Marmota* species evolved more recently. The observed higher diversity of molar shape in *Marmota monax* may reflect their greater adaptive potential due to repeated geographic fragmentation and thus could explain the wide range in habitat climates observed today. Another exception is *Marmota marmota*, whose environment has the highest mean humidity among the studied sciurid species we studied, although most of the observation sites refer to arid environments (see fig. 9 and supplementary fig. 2, Supplementary Material online). In the Alps, this species prefers habitats that match less vegetation and high sun exposure with early snowmelt (Allainé et al. 1994). However, future investigations of tree squirrels (Protoxerini, Sciurinae, and other subfamilies) need to prove the *SCNN1D* loss as a potential ecological adaptation in Sciuridae.

Among Supramyomorpha the loss of *SCNN1D* is restricted to Heteromyidae, Dipodidae, and certain Muroidea. Besides being a potential phylogenetic signal (autapomorphic), the *SCNN1D* loss in Heteromyidae and Dipodidae could be also constrained by the arid environment the investigated species live in (Wilson et al. 2016, 2017). Concerning Muroidea, the *SCNN1D* loss may be a synapomorphic character of Eumuroida, a clade comprising Nesomyidae, Cricetidae, and Muridae. Future studies of Calomyscidae need to prove if the *SCNN1D* loss is characteristic of a more exclusive clade. However, no climatic or dietary signals are evident as our sample comprises species with very diverse diets (omnivorous, herbivorous, insectivorous), habitats, and geographic distributions (Wilson et al. 2017). This indicates that the loss of *SCNN1D* is not a disadvantage for the respective species.

Within Hystricomorpha only *D. patagonum* lost *SCNN1D*, although many members of this suborder inhabit arid environments. The Patagonian mara differs from other caviomorph rodents in reflecting a small ruminant adapted to open grass grasslands. However, a study on the digestive system and metabolic rates of *D. patagonum* shows no significant differences from other caviomorphs (Clauss et al. 2019). Therefore, no plausible explanation for the derived pattern in *D. patagonum* can be provided at this point.

In general, possible geographic patterns (e.g., presence of *SCNN1D* in South American species, absence of *SCNN1D* in East Asian species) are certainly biased by our taxon sampling. For instance, given the fact that the Cricetidae members we investigated are all showing *SCNN1D loss*, it is likely that South American members of this family, which were not included in our study, also show the same pattern. Further, the available GBIF data might be biased as numbers of individual observations vary substantially between species. Nevertheless, the broad geographical distribution of species lacking *SCNN1D—*including the Eumuroida—indicates that the absence of *SCNN1D* does not appear to be a general disadvantage or adaptation to an extreme environmental climate. Rather, our data indicate that the role of δβγ-ENaC in renal sodium and water homeostasis might not be as crucial as that of αβγ-ENaC.

Whether δ-ENaC is regulated by aldosterone, expressed in the mammalian distal nephron, or forms functional δβγ-ENaCs in the kidney remains unknown. None of the analyzed rodent species with a functional *SCNN1D* appears to lack a *SCNN1A* gene coding for α-ENaC (data not shown). Given the efficacy of the RAAS in controlling sodium and water homeostasis across a wide range of mammalian species living in various environments, there does not appear to be any obvious benefit in an additional ENaC isoform with high activity in renal tubules. Further, [Na^+^] in the distal convoluted tubules and cortical collecting ducts are lower than plasma [Na^+^] and, based on the *I*_M_–[Na^+^] relationships observed in this study, differences in the activity between αβγ-ENaCs and δβγ-ENaC appear unlikely under these conditions. This might explain why pseudogenization of *SCNN1D* does not appear to correlate with habitat aridity across Rodentia.

The specific *I*_M_–[Na^+^] relationships might indicate that δβγ-ENaC operates under extracellular [Na^+^] that are equal or greater than plasma [Na^+^]. In humans, RNA and protein expression of δ-ENaC was observed in taste buds, which are exposed to a wide range of dietary [Na^+^], including concentrations that greatly exceed plasma [Na^+^] (Bigiani 2020a). In mice, αβγ-ENaC is involved in attractive salt taste to [Na^+^] lower than plasma [Na^+^] (Chandrashekar et al. 2010; Nomura et al. 2020). In humans, the role of ENaC in salt taste is unclear (Bigiani 2020b) but it was recently suggested that δ-ENaC might be relevant for the detection of [Na^+^] that exceed plasma [Na^+^] (Bigiani 2020b). The functional properties of δβγ-ENaC we observed here would be consistent with this notion. δβγ-ENaC could be involved in mechanisms triggering aversive responses to potentially dangerous [Na^+^] that exceed plasma [Na^+^] and renal urine concentration capacity. Such a secondary role in “danger signaling” might explain the lack of obvious selection pressures maintaining *SCNN1D* purely based on environmental factors. Of note, current understanding of salt taste in rodents is based on studies using mice (Chandrashekar et al. 2010; Nomura et al. 2020) which lack functional δ-ENaC and may have evolved alternative mechanisms for aversive salt taste signaling.

Taken together, the data presented here show that *SCNN1D* is not generally absent across rodents and that pseudogenization appeared independently in different clades. Despite genomic changes such as exon fusions, guinea pig δβγ-ENaC is a functional ion channel which has biophysical and regulatory characteristics that are very similar to those of the human ortholog. Guinea pigs are therefore suitable, commercially available rodent model animals that allow future investigations to shed light on the physiological function of δβγ-ENaC.

## Materials and Methods

### Bioinformatical Analyses

Bioinformatical and phylogenetic analyses investigating the absence or presence of *SCNN1D* genes were performed essentially as has been described previously (Maxeiner et al. 2020). In short, the search query “*SCNN1D* and rodentia” yielded results for some but not all currently annotated rodent genomes. Those in which *SCNN1D* was present were consistently flanked by the neighboring genes *UBE2J2* and *ACAP3*. In cases in which *SCNN1D* was not annotated, we used this particular genomic region and performed a sequence alignment to identify potential traces of a not yet annotated or by mutation decaying *SCNN1D* (pseudo-) gene. Information from the latter case were included in a phylogenetic tree based on the otherwise highly conserved exon 6 present and retained in size throughout all *SCNN1* genes (cf. supplementary fig. 1, Supplementary Material online). Additionally, in our evolutionary assessment based on the phylogeny of rodent families reported by D’Elía et al. (2019), we extended our search employing the BLAST tool to search within distinct rodent families with default settings for blast and megablast searches. Supplementary data sheet 1, Supplementary Material online, compiles the information that we retrieved from our search regarding exons sizes etc.

The following sources were used for this study (all last accessed September 2021): NCBI databases (https://www.ncbi.nlm.nih.gov/gene/), BLAST tool (https://blast.ncbi.nlm.nih.gov/Blast.cgi), Expasy sequence translation (https://web.expasy.org/translate/), multiple sequence alignment tools MultAlin (Corpet 1988; http://multalin.toulouse.inra.fr/multalin) and Clustal Omega (https://www.ebi.ac.uk/Tools/msa/clustalo/), DNA/RNA GC Content Calculator (http://www.endmemo.com/bio/gc.php), graphical codon usage analyser (codon usage, https://gcua.schoedl.de), DNA reverse complement tool (http://reverse-complement.com), and phylogeny tool MEGA X (Kumar et al. 2018).

Images of ENaC structures were based on the cryo-EM-derived structures of human αβγ-ENaC (PDB code 6BQN) (Noreng et al. 2018) and the extracellular domain of human αβγ-ENaC (PDB code 6WTH) (Noreng et al. 2020). Structures were visualized with open-source PyMol (Version 2.0, Schrödinger, LLC).

To investigate the geographical distribution of rodent species and correlate functional *SCNN1D* with habitat aridity, positive occurrence data for Rodentia were downloaded from the GBIF (2020). GBIF is an international network and data infrastructure that provides open access to over 60,000 species-location data sets, from historical museum specimens and collections through to georeferenced smartphone photographs, that are combined using common data standards into a singular database of almost 2 billion occurrence records that can be queried. We extracted all positive occurrence data for Rodentia, excluding those from zoological institutions (accessed September 24, 2020, for the specific download information, see GBIF [2020]). This initial data set was restricted to georeferenced observations (i.e., those with a known latitude and longitude). We further excluded occurrence data observed prior to 1900 due to the potential increase in misidentification. We limited the data set to the 51 species for which genomes descriptions are available (cross-referencing potential differences in taxonomic naming, see table 1 for discrepancies). Species were cross referenced against the Global Invasive Species Database (GISD, available at http://www.iucngisd.org/gisd/, last accessed September 24, 2020), which is managed by the Invasive Species Specialist group of the IUCN, with eight species deemed to be invasive (see table 1). These were excluded due to their likely occurrence beyond their natural distribution within the GBIF database, biasing further analyses. The resulting data set consisted of 223,392 geolocated observations for 43 species. To check the quality of the data set, occurrence locations were mapped for each species and cross-checked against their natural distributions according to (Wilson et al. 2016, 2017). Records falling outside of known species’ ranges were removed along with observations that mapped onto the marine environment, resulting in a cleaned data set of 218,410 observations (supplementary figs. 2–5, Supplementary Material online). Global aridity data were accessed from the Global Aridity Index and Potential Evapotranspiration Climate Database v2, which is supported by the CGIAR Consortium for Spatial Information (accessed September 24, 2020, available at https://doi.org/10.6084/m9.figshare.7504448.v3). The aridity index estimated as the ratio of mean annual precipitation to mean annual reference evapo-transpiration, and is provided as a 30 arc-second resolution (∼30 m) raster giving the 30-year average for the period 1970–2000 modeled using the WorldClim Global Climate Data (for details on methodology, see Trabucco and Zomer [2018] and https://cgiarcsi.community/2019/01/24/global-aridity-index-and-potential-evapotranspiration-climate-database-v2/). For each rodent observation, we extracted an aridity index taken as the median of all aridity surface values encompassed within radius of 0.02° of the observation (∼2 km), to limit potential anomalies in microclimates.

### Nucleic Acid Isolation

Guinea pigs (*C. porcellus*; “Hartley GP, retired breeders”) were purchased from Charles River Laboratories (Sulzfeld, Germany). All animal handling was approved of and followed the standards set by the animal welfare and ethics committee of Saarland University and the local authorities. Tissue samples for nucleic acid extraction were collected in TRIzol reagent for RNA extraction (Invitrogen, Carlsbad, CA) and DNA extraction was performed from blood samples using the Quick-DNA MiniPrep Plus extraction kit (Zymo Research Europe GmbH, Freiburg, Germany) according to the manufacturer’s protocol. RNA extraction strategies involved traditional TRIzol/chloroform extraction with 2-propanol precipitation or purification by column resin using the Direct-zol RNA MiniPrep Plus kit (Zymo Research Europe GmbH). Nucleic acids were quantified using a NanoDrop One spectrophotometer (VWR International GmbH, Darmstadt, Germany) and stored until further use at −80 °C.

### 5′RACE Experiment

In order to determine the transcriptional start of all four guinea pig *SCNN1* genes, cDNA was generated from testis and brain RNA using the SMARTer RACE 5′/3′ Kit (Takara Bio USA, Inc., Mountain View, CA) and followed the manufacturer’s protocol generating amplicons using a nested primer approach. All gene specific oligomers were purchased from IDT (Coralville, IO). The 5′RACE cloning strategy aimed at the inclusion of at least a single exon/exon junction to rule out artifacts of potential transcriptional start sites from contaminations of genomic DNA. All amplicons were cloned into a pRACE mini vector using the In-Fusion HD Cloning Kit (Takara Bio USA, Inc.; Mountain View, CA) and validated by Sanger sequencing (ATGC-Lab, MPI for Experimental Medicine, Göttingen, Germany).

### Cloning of Guinea Pig *SCNN1* Genes

Based on current sequence information (NCBI), primers were designed for PCR amplification aiming at a full coverage of the potential guinea pig *SCNN1D* gene. Due to the relatively high GC content of the genomic region, a combination of polymerases has been applied to yield in a full coverage of the gene, such as PrimeSTAR HS DNA Polymerase, SeqAmp DNA Polymerase (both: Takara Bio USA, Inc.), or Q5 High-Fidelity 2X Master Mix (New England Biolabs GmbH, Frankfurt, Germany). The products were subcloned by Gibson assembly strategies using the NEBuilder HiFi DNA Assembly Cloning Kit (New England Biolabs GmbH). Based on bioinformatical analyses of the *SCNN1A*, *B*, and *G* genes as well as our own results of the *SCNN1D* gene sequencing effort, the coding sequence was inferred and mini genes custom-synthesized (gblocks from IDT, Leuven, Belgium) with an optimized codon usage applying to the *Xenopus* expression system. Subsequently, all mini genes were cloned into an *Eco*RI/*Xba*I linearized pTNT vector (Promega Corporation, Madison, WI).

### Sequence Deposition

The sequence information of all guinea pig *SCNN1* 5′RACE sequences (*SCNN1A*, *SCNN1B*, *SCNN1G*, and *SCNN1D*) as well as the full coverage of the guinea pig *SCNN1D* gene were deposited with NCBI GenBank using the following accession numbers: *SCNN1A* 5′RACE product (MN187466); *SCNN1B* 5′RACE product (MN187463); *SCNN1G* 5′RACE product A (MN187464); *SCNN1G* 5′RACE product B (MN187465); *SCNN1D* 5′RACE short version (MN187468); *SCNN1D* 5′RACE long version (MN187469); *SCNN1D* gene (MN187539).

### Plasmids and cRNA Synthesis

The DNA coding sequences for guinea pig, human, and *Xenopus laevis* α-, β-, γ-, and δ-ENaC subunits were cloned into the pTNT expression vector (Promega Corporation). Plasmids were transformed into *Escherichia coli* (K12, DH5α) and isolated using QIAprep Spin Miniprep kit (Qiagen, Manchester, United Kingdom). Plasmid cDNAs for human ENaC subunits were linearized with FastDigest *Bam*HI (ThermoFisher Scientific, Gloucester, United Kingdom) per manufacturer’s instructions. The guinea pig ENaC subunits were not linearized due to presence of restriction sites within the coding sequences. ENaC subunit cRNAs were generated by in vitro transcription with T7 RNA polymerase (Ribo-MAX large-scale RNA production system, Promega Corporation) in accordance with the manufacturer’s instructions. The ENaC subunit cRNAs were then diluted with diethyl pyrocarbonate (DEPC)-treated water to a final concentration of 10 ng/µl for human and 5 ng/µl for guinea pig ENaCs for two-electrode voltage-clamp recordings. For patch-clamp recordings, guinea pig ENaC cRNA was diluted to 20 ng/µl per subunit.

### Isolation of *Xenopus* Oocytes and cRNA Injection

The experimental procedures were approved by the Animal Welfare and Ethical Review Body at Newcastle University (project ID 630). *Xenopus laevis* ovaries were purchased from the European *Xenopus* Resource Centre (EXRC, Portsmouth, United Kingdom). Ovary lobes were manually dissected with forceps before being incubated and rocked (40 rpm) for 90 min in Oocyte Ringer Solution II (ORII; 82.5 mM NaCl, 2 mM KCl, 1 mM MgCl_2_, 5 mM 4-(2-hydroxyethyl)-1-piperazineethanesulfonic acid [HEPES], pH 7.5) containing 2.6 mg/ml collagenase A (Roche, Welwyn Garden City, United Kingdom) in order to remove the follicular cell layer. Oocytes were then washed six times with ORII and washed a further six times with Modified Barth’s Solution (MBS; 88 mM NaCl, 1 mM KCl, 0.41 mM CaCl_2_, 10 mM HEPES, 2.4 mM NaHCO_3_, 0.33 mM Ca(NO_3_)_2_, 0.82 mM MgSO_4_, 20 µg/ml gentamycin, pH 7.5). Stage V and VI oocytes were manually selected and placed in MBS. Oocytes were injected (Nanoject automatic oocyte injector, Drummond Scientific, Broomall, PA) with 13.8 nl of αβγ- or δβγ-ENaC cRNA and incubated at 16 °C for 16–24 h in low sodium N-methyl-d-glucamine-oocyte Ringer Solution (NMDG-ORS; 80 mM NMDG, 10 mM NaCl, 1 mM KCl, 2 mM CaCl_2_, 2.5 mM Sodium pyruvate, 5 mM HEPES, 20 µg/ml gentamycin, pH 7.4). For patch-clamp recordings, oocytes were incubated for 2–7 days.

### Two-Electrode Voltage-Clamp Recordings

Oocytes were clamped at a holding potential of −60 mV using a Warner oocyte voltage clamp amplifier (OC725B/C Warner Instruments, Hamden, CT). Whole cell transmembrane current signals (*I*_M_) were filtered at 1 kHz and were recorded using a strip chart recorder. Oocytes were superfused at room temperature with Oocyte Ringer Solution (ORS; 90 mM NaCl, 1 mM KCl, 2 mM CaCl_2_, 5 mM HEPES, pH 7.4) at a perfusion speed of 3–5 ml/min, unless otherwise stated. The application of amiloride (Alfa Aesar, Heysham, United Kingdom) was used to determine the fraction of the *I*_M_ that was generated by ENaC (amiloride-sensitive current, Δ*I*_ami_).

### Patch-Clamp Recordings

Patch-clamp recordings were performed using the cell-attached configuration as previously described (Wichmann et al. 2019). Mechanically devitellinized oocytes were placed in a recording chamber filled with bath solution (145 mM KCl, 1.8 mM CaCl_2_, 10 mM HEPES, 2 mM MgCl_2_, 5.5 mM glucose, pH 7.4). Borosilicate glass capillaries were used to generate patch-pipettes (6–10 MΩ resistance) by employing a two-stage puller (PP83, Narishige, London, United Kingdom). The patch-pipettes were then heat polished before being filled with pipette solution (145 mM NaCl, 1.8 mM CaCl_2_, 10 mM HEPES, 2 mM MgCl_2_, 5.5 mM glucose, pH 7.4). A LM-PC patch-clamp amplifier (List-Medical, Darmstadt, Germany) was used to amplify current signals which were low-pass filtered at 100 Hz (Frequency Devices, Haverhill, IL). Current signals were recorded at 2 kHz using an Axon 1200 interface with Axon Clampex software (Axon Instruments, Foster City, CA). All experiments were performed at room temperature. Single channel analysis was performed using Clampfit 10.7 software (Molecular Devices, Wokingham, United Kingdom).

### Chemicals and Reagents

Amiloride hydrochloride, CaCl_2_, Ca(NO_3_)_2_, NaHCO_3_, MgCl_2_, and MgSO_4_ were from Alfa Aesar (Heysham, United Kingdom). α-chymotrypsin, gentamycin (10 mg/ml), DEPC, glucose, NaCl, N-methyl-d-glucamine (NMDG), and sodium pyruvate were from Sigma Aldrich (Dorset, United Kingdom). Dimethyl sulfoxide (DMSO), HEPES and KCl were purchased from ThermoFisher Scientific (Gloucester, United Kingdom). Stock solutions of 100 mM amiloride were made in DMSO and stored at 4 °C. Stock solutions of α-chymotrypsin (2 mg/ml) were freshly made in ORS on the day of experiments, kept on ice, and used within 4 h.

### Data Analysis and Statistics

#### Electrophysiology

Data are presented as means ± SEM and “*n*” represents the number of experiments performed. Each experimental approach was completed across two to three oocyte donors. Statistical analysis was performed using GraphPad Prism (v8.0.1; GraphPad Software Inc., San Diego, CA). The D’Agostino-Pearson omnibus normality test was used to assess whether data had a Gaussian distribution. Data sets with Gaussian distribution were analyzed using the two tailed Student’s *t*-test. In addition, a Welch’s correction was performed if the variances were not equal. Normally distributed multiple groups were analyzed using an ordinary one-way ANOVA with post hoc Tukey’s multiple comparison test. Data sets that did not follow Gaussian distribution were analyzed using the two tailed Mann–Whitney *U* test. A Kruskal–Wallis test with Dunn’s multiple comparison test was used for the analysis of nonparametric multiple groups. Data in figure 8*C* and *E* were each fitted to a simple linear regression model and regression lines were compared using ANCOVA. All figures were assembled and finalized using Inkscape (v0.92.3).

#### Correlation of Functional *SCNN1D* with Habitat Aridity

For each rodent observation, an aridity value was extracted as the median of all aridity surface values encompassed within a radius of 0.02° of the observation (∼2 km). To test whether gene expression explains any variation in habitat aridity, gamma error distribution (logarithmic link function) nested generalized linear mixed models were constructed with species within clade as nested random effects and with or without gene presence as a categorical fixed effect. Models were compared with test whether inclusion of gene presence significantly improved model fit. Analyses were conducted in R programming language (R Core Team 2019) with models constructed using the package “lme4” (Bates et al. 2015), model comparison conducted using “lmerTest” (Kuznetsova et al. 2017), and aridity data handled using the package “raster” (Hijmans).

## Supplementary Material

Supplementary data are available at *Molecular Biology and Evolution* online.

## Supplementary Material

msab271_Supplementary_Data
