# Corrigendum to: Two Functional Epithelial Sodium Channel Isoforms Are Present in Rodents despite Pronounced Evolutionary Pseudogenization and Exon Fusion

**DOI:** 10.1093/molbev/msab328

**Published:** 2021-12-13

**Authors:** Sean M Gettings, Stephan Maxeiner, Maria Tzika, Matthew R D Cobain, Irina Ruf, Fritz Benseler, Nils Brose, Gabriela Krasteva-Christ, Greetje Vande Velde, Matthias Schönberger, Mike Althaus

*Molecular Biology and Evolution*, msab271, https://doi.org/10.1093/molbev/msab271

Upon the original publication of this article, we noted an error in the Supplementary Data. The alignment of SCNN1 protein sequences in Supplemental Data 2 was performed using the current GenBank entry for guinea pig delta-ENaC (Gene ID: 100714892). As reported in the manuscript, our sequencing efforts identified a change in the very C-terminal protein sequence (deposited on GenBank: MN187539) with respect to the current entry (Gene ID: 100714892). The corrected version was used in all experiments and has now been updated in the alignment in order to avoid confusion.

