# Supplementary material for: Two Functional Epithelial Sodium Channel Isoforms Are Present in Rodents despite Pronounced Evolutionary Pseudogenization and Exon Fusion
Source: Mol Biol Evol. 2021 Sep 7;38(12):5704–25. doi: 10.1093/molbev/msab271 (PMC8662647; doi:10.1093/molbev/msab271)
Supplement: msab271_Supplementary_Data [file msab271_supplementary_data.zip › Supplemental Data_Corrected_251021_4pm.pdf]

Supplemental Data 1

Overview of *SCNN1D* exon 6 sequences of all species used in the phylogenetic tree in Supplemental Figure 1. The following sequences derive from genomic information available for the respective rodent species deposited in Genbank (NCBI). Not all species' genomes have been annotated upon submission.
Individual sequences were analyzed by sequence comparison of neighbouring species. Along with our suggestion to standardize the comparison of *SCNN1* genes (this paper), we compiled this list of *SCNN1D* exon 6 being present in all species analyzed. The general base count of exon 6 is 164. Some species that have lost a functional *SCNN1D* gene still retain sequences with a degree of similarity to the formerly functional *SCNN1D* gene (highlighted in yellow). Consequently, some of these examples of formerly functional exon 6 sequences have a base count deviating from 164. The incorporation of those exon 6 versions is justified and highlights the evolutionary most recent loss in distinct branches within the rodent order.

Exon 6 sequences are listed alphabetically in FASTA format.

>Allactaga bullata
GAATCAGCCTGGTCCTCAAGGCTGAACAGTAGGATCGCCTCCCTCTGCTGTCTTCAGAGACTGGA
ATCAAGATCATGGTTCATGGGCACAACAGCACATCTTTCCTGGAACACTATGGCTTCAGCATTCG
GCCAGGGACCCAGACCACCATAGGAATCTGAGAG

>Aplodontia rufa
GGATCAGCCTGGTCGTCAGGGCCAAGCAGCAGGATCACCTCCCTCTGCTATCCACTGAGGCCGGT
ATCAAGGTCATGGTTCACGGGCACAACCACACACCCTTCCTAGAGCACCTGGGTTTCAGTATCCG
ACCAGGCACTGAAACCACCATTGGCATCAGAGAG

>Capromys pilorides
AACTTAGCCTGGTGCTCAGGACAGAGCCCCAAGTGGGGTTCCCTCTGTTGTCTACGGAGGCCAAT
ATCAAGGTCATGATCCACAGACACAATCACACGCCCTTCTTGGAGCACCAAGGGTTCAGCATACG
GCCTGGGACTGAGACCACCATTGGCATCCGAGAG

>Castor canadensis
GGATCAGCCTGGTCCTCAGAGCTGAGCAGCAGTATCACCTCCCTTTGCTGTCCGAGGAGACGGGC
ATCAAGATCATGATTCACGGGCACAACCACACACCCTTCCTGGAACATCGTGGCTTCAGCATCCG
CCCAGGGACGAAGACCACCATTGGCATCAGAGAG

>Cavia_porcellus
AGATCAACCTGATCCTCAGGACAGAACCGCGAGTGGGGCTCCCTCTGCTGTCTACCGAGGCCGAC
ATTAAGGTCATGATTCACGGACACAACCATACGCCCTTCCTGGAGCACCGGGGATTCAGCGTCCG
GCCTGGGACTGAGACCACTATCGGCATCCGCGAG

>Cavia_tschudii
AGATCAACCTGATCCTCAGGACAGAACCGCGAGTGGGGCTCCCTCTGCTGTCTACCGAGGCCGAC
ATTAAGGTCATGATTCACGGACACAACCATACGCCCTTCCTGGAGCACCGGGGATTCAGCGTCCG
GCCTGGGACTGAGACCACTATCGGCATCCGCGAG

```
>Chinchilla lanigera
AGATCAGCCTGGTCCTCAGGACAGAGCCTCAAGTGGAGCTCCCTCTGCTGTCTACGGAGGCCGAC
ATCAAGGTCATGATTCACGGACACAACCACACGCCCTTCCTGGAGCACAGAGGGTTCAGCCTTCG
GCCTGGGACTGAGACCACCATCGGCATCCGAGAG

>Cricetomys gambianus
GGATCAGCTGGTCTTCAAGACTAAAAAGCAAGACCACCTCCCTCTTTTGTCCTTGGAGGCTGGAA
TCTAGGTCATGGTTTATAGGTCCAATCAGATATCCTTCTGGAGCAACAGTGACTTCAGCATCCCA
CCAGGGATTCATATTGCCATTGGAATCCAGAG

>Cricetulus griseus
GAATCAGCCTAGTCCTCAGGGCTAAAAGGCAAGAACATCTCCTTCTTTTGTCCTTGGAGGCTGGA
ATCAAGGTCATCATTCATAGGTTCAACCACACATCCTTCTAGAATACAATGGCTCCTTCTGACCA
GGGACTCAGAATACCACTGGAATTCGAGGG

>Ctenodactylus gundi
GGATCAGCCTGATCCTCAGGGCCAGGCCACAGAGTCACCTCCCTTTGCTGTCCACTGAGGCCGGC
ATCAAGGTCACAATCCATGGACATGACCACACGCCCTTCCTGGAGCATCGTAGCTTTGAGATCCG
GCCAGGAACCGAGACCACCATCGGCATTCGGGAG

>Ctenomys sociabilis
AGATCAGCCTGGTGCTCAGGACAGAGCCCCAAGTGGGGTTCCCTCTGTTGTCTACGAAGGCAGAC
ATCAAGGTCATGATCCACGGACACAATCACACGCCCTTCCTGGAGCACCAAGGGTTCAGCATAAG
GCCTGGAACTGAGACCACCATTGGCATCCGAGAG

>Cuniculus paca
AGATCAACCTGGTCCTCAGGACAGAGCCGCGAGTGGGGCTCCCTCTGCTGTCTACCGAGGCCGAC
ATTAAGGTCATGATTCACGGACACAACCACACGCCCTTCCTGGAACACCGAGGATTCAGCATCCG
ACCTGGCACTGAGACCACCGTCGGCATCCGCGAG

>Cynomys gunnisoni
GTATCAGTCCGGTCCTCAGGACTGAGCTGCAGCAGTATCTCACGCTGCTGTCCACCGAGGCTGGT
GTCAAGGTCATGGTTCACGGGCACAACCACACGCTCTTATTAGAGCACCAGGATTTCAGTATCCG
GCCAGGGATGGAGACCACAATTGACATCCGAGAG

>Dasyprocta punctata
AGATCAACCTGGTCCTCAGGACAGAGCCGCGAGTGGGGCTCCCTCTGCTGTCTACCGAGGCCGAC
ATTAAGGTCATGATTCACGGACACAACCACACGCCCTTCCTGGAGCACGGAGGGCTCAGCATTCG
GCCTGGGACTGAGACCACCATCGGCATCCGAGAG

>Dinomys branickii
AGATCAGCCTGGTCCTCAGAACAGAGCCGCGAGTGGAGCTCCCTCTGCTGTCTACCGAGGCCGAC
ATCAAGGTCATGATTCACGGACACAACCACACGCCCTTCTTGGAACACAGAGGGTTCAGTATTCG
GCCTGGGACTGAGACCACCATCGGCATCCGAGAG

>Dolichotis patagonum
AGATCAACCTGATCCTCAGGACAGAGCCGTGAGTGGGGCTCCCTCTGCTGTATACTGAGGCCGAC
ATTAAGGTCATGATTCACAGACACAGTCACACGCCCTTCCTAGAGCACCGAGGGTTCAGCGTCCG
GCCTGGGACTGAGACCACTATAGGCATCCGCGAG
```

```
>Erethizon dorsatum
AGATCAGCCTGGTCCTCAGGACAGAGCCGCAAGTGGGGCTCCCTCTGCTGTCTACCGAGGCCGAC
ATTAAGGTCATGATTCACGGACACAACCACACGCCCTTCCTGGAGCACCGAGGGTTCAGCATCCG
GCCTGGGACTGAGACCACCATCGGCATCAGAGAG

>Glis glis
GGATCAGCCTGGTCCTCAGGGCTAAGCAGCAGGATCACATGCCTCTACTCTCCACCGAGGCCGGC
ATCATGGTCATGGTTCATGGGCACAACCATACACCCTTCCTGGAGCACCTGGGCTTCAGCATCCG
GCCTGGGACTGAGACCACCATTGGCATCCGAGAG

>Grammomys surdaster
AGATCAGTCTGGTCCTCAAGGCTAAAAGGCAAGATCACTTCCCTCTTTTGTCCTTAGAGGCTGGA
ATCAAGGTCAAGGTTCATAGCTACAACACACTCTTTTGGAGCACAGTGACTTCATACTACCACTA
GACTCATACTACCATTAGAATTCGAGAG

>Graphiurus murinus
GGATCAGCCTGGTCCTCAGGGCTGAACAGCAGGATCACCTGCCTCTGCTCTCCACCGAGGCCGGC
ATCAGGGTCATGGTTCACGGGCGCAACCACACGCCCTTCCTGGAGCACCTGGGCTTCAGCATCCG
ACCTGGGACAGAGACCACCATCGGCGTCCGAGAG

>Heterocephalus glaber
AGATCAGCCTGGTCCTCAGGACACAGACGCAAGTGGTGCTCCCCCTGCTGTCCACCGAGCCTGAC
ATCAAGGTCATGATTCACGGGCACAACCACACGCCCTTCCTGGAGCCCCGAGGCTTCAGCATCCG
GCCTGGGCTGGAGACCACCATCGGCATCCGAGAG

>Hydrochoerus hydrochaeris
AGATCAACCTGATCCTCAGGACAGAGCCGCGAGTGGGGCTCCCTCTGCTGTCTACCGAGGCCGAC
ATTAAGGTCATGATTCACGGACACAACCACACGCCCTTCCTGGAGCATCGAGGGTTCAGCATCCG
GCCTGGGACTGAGACCACTATAGGCATCCGCGAG

>Hystrix cristata
AGATCAGCTTGGTCCTCAGGACAGAGCCGCAAGTGGGGCTCCCTTTGCTGTCCACGGAGGCCGGC
ATCAAGGTCATGATTCATGGGCACAAGCACACCCCCTTCCTGGAGCACCGAAGCTTCAGCATCCG
GCCTGGGACTGAGACCACCATCAGCATCCGCGAG

>Ictidomys tridecemlineatus
GTATGAGTCCGGTCCTCAGGACTGAGCTGCAGCAGTATCTCACGCTGCTGTCCACCGAGGCTGGT
GTCAAGGTCATGGTTCACGGGCACAACCACACGCTCTTATTAGAGCACCAGGATTTCAGTATCCG
GCCACAGTTGACATCCGAGAG

>Jaculus jaculus
GAATCAGCCTGGTCCTCAGGGCTGAAGAGCAGGATCACCTCTCTCTGCTGTCTACAGAGACTGGA
ATCAAGGTTATGGTTCATGGACACAATACACCTTTCCTGGAACACTATGGCTTCAGCATTCAGCC
AGAGACCCAGACCACTATAGGAATCCAAGAG

>Marmota_himalayana
GTATCAGTCTGGTCCTCAGGACTGAGCTGCAGCAGTATCTCACGCTGCTGTCCACCCAGGCTGGT
ATCAAGGTCATGGTTCATGGGCACAACCACACGCTCTTATTAGAGCACCAGGATTTCAGTATCTG
GCCAGGGATGGAGACCACAGTCGACATCCAAGAG
```

>Marmota_monax
GTATCAGTCTGGTCCTCAGGACTGAGCTGCAGCAGTATCTCACGCTGCTGTCCACCCAGGCTGGT
ATCAAGGTCATGGTTCATGGGCACAACCACACGCTCTTATTAGAGCACCAGGATTTCAGTATCTG
GCCAGGGATGGAGACCACAGTTGACATCCAAGAG

>Marmota_marmota
GTATCAGTCTGGTCCTCAGGACTGAGCTGCAGCAGTGTCTCACGCTGCTGTCCACCCAGGCTGGT
ATCAAGGTCATGGTTCATGGGCACAACCACACGCTCTTATTAGAGCACCAGGATTTCAGTATCTG
GCCAGGGATGGAGAGCACAGTTGACATCCAAGAG

>Mastomys coucha
AGATCAGCCTGGTCCTCAAGGCTCCCTCTTTTGTTCTTGGAGGCTGGAGTCAAGGTCATGGTTCA
TAGTTACAACACACTGTTCTGGAGCACAGTGGCTTCATACTAACATTGGACTCATACTACCATTG
GAATTCAAGAG

>Mesocricetus auratus
GGATCAGCCTAGTCCTCAGGGCTAAAAGGCAAGAACATCCCCCTCTTTTGTCCTTGGAGACTGGA
ATCAAGGTCATCGTTCATAGGAACAACCACACACCCTTCTAGAACACAATGGCTTCAGCTTCTGG
CCGGGGACTCAGAATACCATTGGAATTCAAGGG

>Microtus ochrogaster
GGATCAGCCTAGTCCTCGGGGATAAAAGGCAAGATCATCTCCTTCTTGGAAGGATTTCAGACCTT
GGAGACTGGAATCAAGGACATGGTTCACAACTACACGCACCCTTCTAGAATGCAGTGGCTTCGGT
TTCTGACCAGGGACACAGAATACCATTAGAATTTGAGAG

>Mus_caroli
AGATCAGCCCGGTCCTCAAGGCTCCCTCTTTTGTCCTTGGAGGCTGGAATCAAGGTCATGGTTCG
TAGCTACAACAGCACGCTGTTCTGGGGCACAGTGGCTTCATACTCACATTGGAGTCATACTACCA
TTGGAGTTCAAGAG

>Mus_musculus
AGATCAGCCTGGTCCTCGAGGCTCCCTCTTTTGTCCTTGGAGGCTGGAATCAAGGTCATGGTTCG
TAGCTACAACAACACGCTGTCCTGGGGCACAGTGGCTCCATACTACCATTGGAGTTCAAGAG

>Mus_pahari
AGATCAGCATGGTCCTTGAGGCTCCCTCTTTTGTCCTTGGAGCCTGGAATCAAGGTCATGGTTCA
CTGCTACAACACACTGTTCTGGAGCACAGTGGCTTCATACTAACATTGGAGTCACACTACCATTG
GAATTCAAGAG

>Muscardinus avellanarius
GGATCAGCTTGGTCCTCAGGGCTGAGCAGCAGGATCACCTGCCTCTGCTCTCCACCGAGGCCGGG
ATCAAGGTCATGGTTCATGGGCACAACCACACACCCTTCCTGGAGCACCTGGGCTTCAGCATCCG
GCCTGGGACTGAGACCACCATCGGCATCAGAGAG

>Myocastor coypus
AGATTAGCCTGGTGCTCAGGACAGAGCCCCAAGTGGGGTTCCCTCTGTTGTCTACGGAGGCCGAT
ATCAAGGTTATGATCCACCAACACAATCACACGCCCTTCTTGGAGCACCAAGGGTTCAGCATACG
GCCTGGGACTGAGACCACCGTTGGCATTCGAGAG

>Nannospalax galili
GAATCAGCCTGGTCCTCAGGACTGAGCAGCAAGATCACCTCCCTCTTTTGTCCATGGAGGCTGGA
ATCAAGGTCATGGTTCATGGGCACAACCACACACCTTTCCTGGAACACAGTGGCTTCAACATCCG
GCCAGGGACTCAGACTACCATTGGAATTCGAGAG

>Octodon degus
AGATCAGCCTGGTGCTCAGGACAGAGCCCCAAGTGGGGTTCCCTCTGTTGTCTACGAAGGCGGAC
ATCAAGGTCATGATCCACGGACACAATCACACTCCCTTCCTGGAGCACCGAGGGTTCAGCGTACG
GCCTGGGACTGAGACCACCATTGGCATCCGAGAG

>Pedetes capensis
GGATCAGCCTGATACTCAGGGCTGAGCGGCAGGACCACTTCCCGCTGCTGTCTGCAGAGGCTGGC
GTCAAGGTCACGGTCCATGGGCACAACCACACGCCCTTCCTGGAGCACCACGGCTTCAGCATCCG
GCCAGGGACGGAGACCACCATTGGCATCAGAGAG

>Petromus typicus
AGATCAGCCTAGTCCTCAGGACACAGACACAAGTGGGGCTCCCTCTGCTGTCCACTGAAGCTGAC
ATCAAGGTCATGATTCACGGGTACAACCACACGCCTTTTCTGGAGCACGGAGGCTTCAGCATTCG
GCCTGGGACTGAGACTACCATCAACATCCGAGAG

>Peromyscus_leucopus
GGATCAGCCTAGTCCTCAGAGCTAAAAGGCAAGATCATCTCCCTCTTTTGTTCTTGGAGGCTGGA
CTCAAGGTCATGGTTCATAGGTACGATCACACACCCCTCTAGAAAACAGTGGCTTTAGCTTCTGA
CCAAGGACTCAGAATACCATTGGAATTCTAGAA

>Peromyscus_maniculatus
GGATCAGCCTAGTCCTCAGAGCTAAAAGGCAAGATCATCTCCCTCTTTTGTCCTTGGAGGCTGGA
CTCAAGGTCATGGTTCATAGGTACGATCACACACCCTTCTAGAAAACAGTGGCTTTAGCTTCTGA
CCAAGGACTCAGAATACCATTGGAATTCTAGAA

>Rattus_norvegicus
AGATCAACTTGATCCTCCAGGCTAAAATGCAAGGACACCTCTCTCTTCTGCGGTTGGAGGCTGGA
ATTGAGGTCATGGTTCACAGCTACAACACACTCTTCTGGAGCACAAGTGACTTCATATTGCCGTT
GGACTCATGCTACCGTTGGAATTCGAGAG

>Rattus_rattus
AGATCAACTTGATCCTCCAGGCTAAAATGCAAGGACACCTCTCTCTTCTGTGGTTGGAGGCTGGA
ATCGAGGTCATGGTTCACAGCTACAACACACTCTTCTGGAGCACAGTGACTTCATATTACCGTTG
GACTCATGCTACCGTTGGAATTCGAGAG

>Spermophilus dauricus
GTATCAGTCTGGTCCTCAGGACTGAGCTGCAGCAGTATCTCACGCTGCTGTCCACCGAGGCTGGT
GTCAAGGTCATGGTTCATGGGCACAACCACACGCTCTTATTATCCGGCCAGGGATGGAGACCACA
GTTGACATCCAAGAG

>Thryonomys swinderianus
AGATCAGCCTGGTCCTCAGGACTCAGACGCAAGTGGGGCTCCCTCTGCTGTCCACCAAGGCTGAC
ATCAAGGTCATGATTCACGGGTACAACCACACGCCTTTTCTGGAGCACCAAGGCTTCAGCATTCG
GCCCGGGGCTGAGACCACCATCAGCATTAGAGAG

```
>Urocitellus parryii
GTATCAGTCTGGTCCTCAGGACTGAGCTGCAGCAGTATCTCACGCTGCTGTCCACCGAGGCTGCT
GTCAGGGTCATGGTTCATGGGCACAACCACACGCTCTTATTAGAGCACCAGGATTTCAGTATCCG
GCCAGGGATGGAGACCACAGTTGACATCCAAGAG

>Xerus inauris
GAATCAGCCTGGTCCTCAGAACTGAGCAACAGCAGCATCTCCCTCTGCTGTCCACCGAGGCTGGG
GTCAAGGTCACGGTCCACAGGCACAACCACACACCCTTCTTAGAGCATCTGGACAGTATCTGGAC
AGTATCTTAGAGTATCAGTATCCGGCCAGGGGACGGAAACCACCGTCAGCATCTAAGAG

>Zapus hudsonius
GAATCAGCCTGGTCCTCAAGGCTGTACAGCAGGATCACCTCCCTCTGCTATCTGCAGAGGCCGGA
ATCAAGATCATAGTTCATGGTCACAACCACACGCCCTTCCTGGAACACTGTGGCTTCAGCATTCG
GCCAGGGACCCAGACCACCATTGGAATCCGAGAG
```

Supplemental Data 2

Alignment of the full α- and δ-ENaC peptide sequences (human and guinea pig).

```
Human_alpha-ENaC            ---------------MEGNKLEEQDSSPPQSTPGLMKGNKREEQGLGPEPAAPQQPTAEE     45
Guinea_pig_alpha-ENaC       ---------------MKGDELKAQGPLPPQPLQGPLKGDKCEQPGLGPEPTAPQQHTEEE     45
Human_delta-ENaC            MAEHRSMDGRMEAATRGGSHLQAAAQTPPRPGP--------------PSAPPPPPKEGHQ     46
Guinea_pig_delta-ENaC       ---------------------------------------------------MPPLERGHR     9

                                                                    Transmembrane domain 1
Human_alpha-ENaC            EALIEFHRSYRELFEFFCNNTTIHGAIRLVCSQHNRMKTAFWAVLWLCTFGMMYWQFGLL     105
Guinea_pig_alpha-ENaC       EALIEFHRSYRELFQFFCNNTTIHGAIRLVCSKHNRMKTAFWAVLWLCTFGMMYWQFALL     105
Human_delta-ENaC            EGLVELPASFRELLTFFCTNATIHGAIRLVCSRGNRLKTTSWGLLSLGALVALCWQLGLL     106
Guinea_pig_delta-ENaC       EKLVEIHTSFGELLTFFCKNTTIHGTIRLVCSSPNRLKKVSWGLLLLGTLGMLYWQLGLL     69

Human_alpha-ENaC            FGEYFSYPVSLNINLNSDKLVFPAVTICTLNPYRYPEIKEELEELDRITEQTLFDLYKYS     165
Guinea_pig_alpha-ENaC       FGEYFSYPVSLNINLNSDKLVFPAVTVCTLNPYRYKEIKEQLRELDRITQQTLFDLYNYN     165
Human_delta-ENaC            FERHWHRPVLMAVSVHSERKLLPLVTLCDGNPRRPSPVLRHLELLDEFARENIDSLYNVN     166
Guinea_pig_delta-ENaC       LEQYWRYPVIMAVSIHSERKLFPSVTLCDMNPQRPGSLHYHLEALDAFAQESIYSLYKFN     129

                                     GRIP domain:  P1          P2                     P3
Human_alpha-ENaC            SFTTLVAGSRSRRDLRGTLPHPLQRLRVPPPPHGARRARSVASSLRDNNPQVDWKDWKIG     225
Guinea_pig_alpha-ENaC       ASSTLLAGARSRRSLADTLPYPLQRIPVQPEPRRARS--SDPSSVRDNNPRVDRRDWRVG     223
Human_delta-ENaC            LSKG-------RAALSATVPRH-------EPPFHLDR------EIRLQRLSHSGSRVRVG     206
Guinea_pig_delta-ENaC       FTEG-------RDTPFPNVPDP-------KSPFKLDR------GIQLQWLKHLGNQHKVG     169

                                        P4
Human_alpha-ENaC            FQLCNQNKSDCFYQTYSSGVDAVREWYRFHYINILSRLPETLPSLEEDTLGNFIFACRFN     285
Guinea_pig_alpha-ENaC       FQLCNQNKSDCFYQTSSSGVDGVREWYRFHYINILAQVADTSPSLEEEALGNFIFACRFN     283
Human_delta-ENaC            FRLCNSTGGDCFYRGYTSGVAAVQDWYHFHYVDILALLPAAWEDSHGSQDGHFVLSCSYD     266
Guinea_pig_delta-ENaC       FKLCNSTGGDCFYRTYSSGVTAAQEWYHFHYLDILGLTPTAREDSH---HSHFVLSCRYN     226

Human_alpha-ENaC            QVSCNQANYSHFHHPMYGNCYTFNDKNNSNLWMSSMPGINNGLSLMLRAEQNDFIPLLST     345
Guinea_pig_alpha-ENaC       QAPCTQENYSHFHHPIYGNCYTFNNKNDSSLWMASMPGINNGLSLTLRTEQNDYIPLLST     343
Human_delta-ENaC            GLDCQARQFRTFHHPTYGSCYTVD-----GVWTAQRPGITHGVGLVLRVEQQPHLPLLST     321
Guinea_pig_delta-ENaC       SEDCQAQHFRKFHHPTYGSCYTFE-----GVCTAQHPGITHKINLILRTEPRVGLPLLST     281

Human_alpha-ENaC            VTGARVMVHGQDEPAFMDDGGFNLRPGVETSISMRKETLDRLGGDYGDCTKNGSDVPVEN     405
Guinea_pig_alpha-ENaC       VTGARVTVHGQDEPAFMDDGGFNLRPGVETSISMRKEALDRLGGSYGDCTQDGSDVPVQN     403
Human_delta-ENaC            LAGIRVMVHGRNHTPFLGHHSFSVRPGTEATISIREDEVHRLGSPYGHCTAGGEGVEVEL     381
Guinea_pig_delta-ENaC       EADIKVMIHGHNHTPFLEHRGFSVRPGTETTIGIREDEVRRLGSPYSRCTDGAVSVDVPL     341

Human_alpha-ENaC            LYPSKYTQQVCIHSCFQESMIKECGCAYIFYPRPQNVEYCDYRKHSSWGYCYYKLQVDFS     465
Guinea_pig_alpha-ENaC       LYPSKYTQQVCIHSCFQENMIKQCGCAYIFYPKPKGVEFCDYTNHSAWGYCYYKLQGAFS     463
Human_delta-ENaC            LHNTSYTRQACLVSCFQQLMVETCSCGYYLHPLPAGAEYCSSARHPAWGHCFYRLYQDLE     441
Guinea_pig_delta-ENaC       LYNSSYTRQACLMSCFQQLMVESCSCGYFLHPLPAGAQYCSRARHPAWGHCFYRLHQALE     401

Human_alpha-ENaC            SDHLGCFTKCRKPCSVTSYQLSAGYSRWPSVTSQEWVFQMLSRQNNY------TVNNKRN     519
Guinea_pig_alpha-ENaC       SDSLGCFNKCRKPCNVTIYKLSAGYSRWPSAASQDWIFQMLSLQNNY------TISNKRN     517
Human_delta-ENaC            THRLPCTSRCPRPCRESAFKLSTGTSRWPSAKSAGWTLATLGEQG-----LPHQSHRQRS     496
Guinea_pig_delta-ENaC       THRLSCDSRCPRPCRETSYKLSTTTSRWPSAKSADWVLDVLRGETPSLSLSPGRSQAPRS     461
                                                              Super-exon 11*

                                                Transmembrane domain 2
Human_alpha-ENaC            GVAKVNIFFKELNYKTNSESPSVTMVTLLSNLGSQWSLWFGSSVLSVVEMAELVFDLLVI     579
Guinea_pig_alpha-ENaC       GVAKLNIYFKELNYRTNSESPSVTMVTLLSNLGSQWSLWFGSSVLSVVEMAEFMFDLLVI     577
Human_delta-ENaC            SLAKINIVYQELNYRSVEEAPVYSVPQLLSAMGSLCSLWFGASVLSLLELLELLLDASAL     556
Guinea_pig_delta-ENaC       HEAKVNIFYQELNYHMVDEAPVYSVPQLLSAMGSLWSLWFGSSVLSVIELLELLLDATAL     521

Human_alpha-ENaC            MFLMLLRRFRSRYWSPGRGGRGAQEVASTLASSPPSHFCPHPMSLSLSQPGPAPSPALTA     639
Guinea_pig_alpha-ENaC       TLLMLLRRFRSRYWSPGRGARAAREVACTPPPSLPSRFCAHSA-----------FPTLTA     626
Human_delta-ENaC            TLVLGGRRLRRAWFSWPRASPASGASSIKPEASQMPPPA----GGTSDDPEPS-------     605
Guinea_pig_delta-ENaC       TLLLGFRWLHGAQVSQPETSTVS----VLPCTSPAARGC----GTKIRDPGHP-------     566

Human_alpha-ENaC            PPPAYATLGPRPSPGGSAGASSSTCPLGGP--------        669
Guinea_pig_alpha-ENaC       PPPAYATLSACPPLQGLAGASSAACAPREP--------        656
Human_delta-ENaC            ----GPHLPRVMLPGVLAGVS-AEESWAGPQPLETLDT        638
Guinea_pig_delta-ENaC       ----VVALERVLMEELG---------------------        579
```
