# Supplementary material for: Two Functional Epithelial Sodium Channel Isoforms Are Present in Rodents despite Pronounced Evolutionary Pseudogenization and Exon Fusion
Source: Mol Biol Evol. 2021 Sep 7;38(12):5704–25. doi: 10.1093/molbev/msab271 (PMC8662647; doi:10.1093/molbev/msab271)
Supplement: msab271_Supplementary_Data [file msab271_supplementary_data.zip › Supplemental Figures.pdf]

Supplemental Figure 1

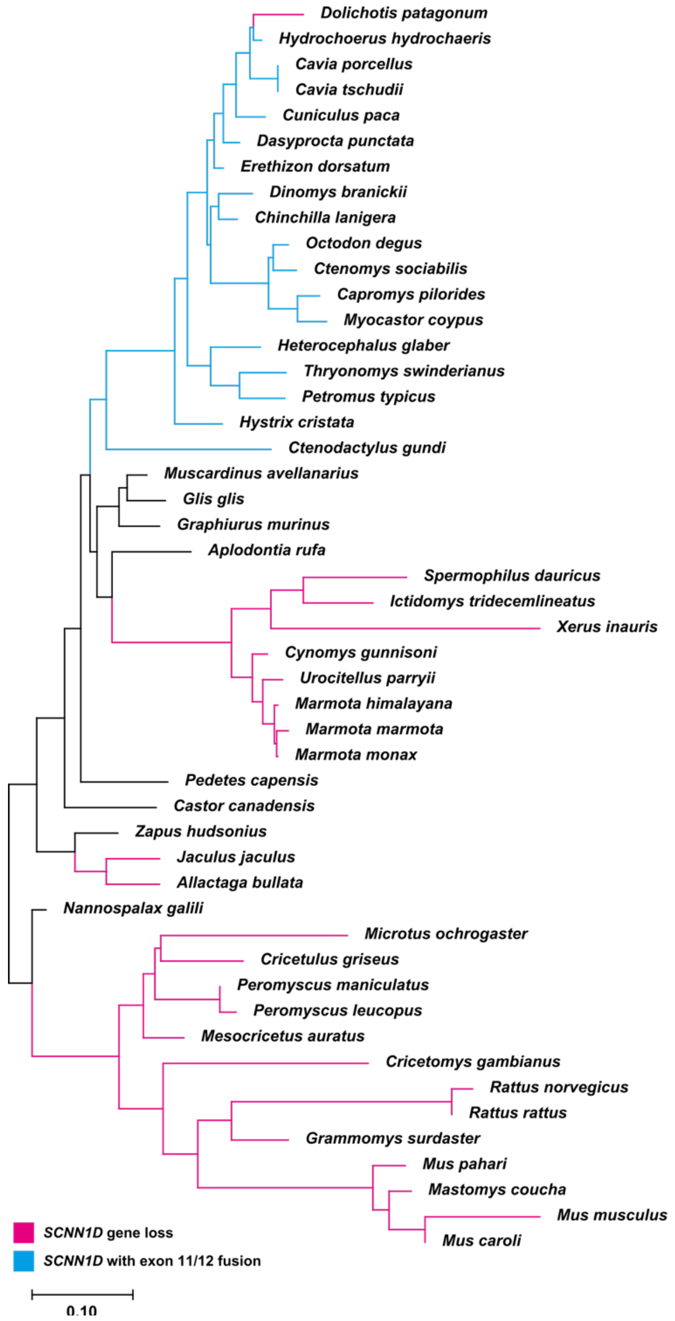

Supplemental Figure 1. Phylogenetic tree of *SCNN1D* genes in rodents

The evolutionary history was inferred using the Neighbour-Joining method [1]. Exon 6 sequences of potentially functional *SCNN1D* genes as well as those of mutated or decaying sequences that could be identified by sequence comparison were used. The optimal tree with the sum of branch length = 3.91250251 is shown. The tree is drawn to scale, with branch lengths in the same units as those of the evolutionary distances used to infer the phylogenetic tree. The evolutionary distances were computed using the Maximum Composite Likelihood method [3] and are in the units of the number of base substitutions per site. The differences in the composition bias among sequences were considered in evolutionary comparisons [4]. This analysis involved 49 nucleotide sequences. All ambiguous positions were removed for each sequence pair (pairwise deletion option). There were a total of 199 positions in the final dataset. Evolutionary analyses were conducted in MEGA X [5][6]. Relationship of sequences

that are incorporated in potentially functional *SCNN1D* genes are depicted in cyan, sequences which are part of mutated/decaying *SCNN1D* genes are depicted in magenta. Interestingly, this tree based on exon 6 is in most parts consistent with current rodent phylogeny [7] (Supplemental Data 2, Supplemental Spreadsheet).

1. Saitou N. and Nei M. (**1987**). The neighbor-joining method: A new method for reconstructing phylogenetic trees. *Molecular Biology and Evolution* **4**:406-425.
2. Felsenstein J. (**1985**). Confidence limits on phylogenies: An approach using the bootstrap. *Evolution* **39**:783-791.
3. Tamura K., Nei M., and Kumar S. (**2004**). Prospects for inferring very large phylogenies by using the neighbor-joining method. *Proceedings of the National Academy of Sciences* (*USA*) **101**:11030-11035.
4. Tamura K. and Kumar S. (**2002**). Evolutionary distance estimation under heterogeneous substitution pattern among lineages *Molecular Biology and Evolution* **19**:1727-1736.
5. Kumar S., Stecher G., Li M., Knyaz C., and Tamura K. (**2018**). MEGA X: Molecular Evolutionary Genetics Analysis across computing platforms. *Molecular Biology and Evolution* **35**:1547-1549.
6. Stecher G., Tamura K., and Kumar S. (**2020**). Molecular Evolutionary Genetics Analysis (MEGA) for macOS. *Molecular Biology and Evolution* (https://doi.org/10.1093/molbev/msz312).
7. D'Elía G., Fabre P.H., Lessa E.P. (**2019**). Rodent systematics in an age of discovery: Recent advances and prospects. *Journal of Mammalogy* 1**00**(3): 852-871

Supplemental Figure 2

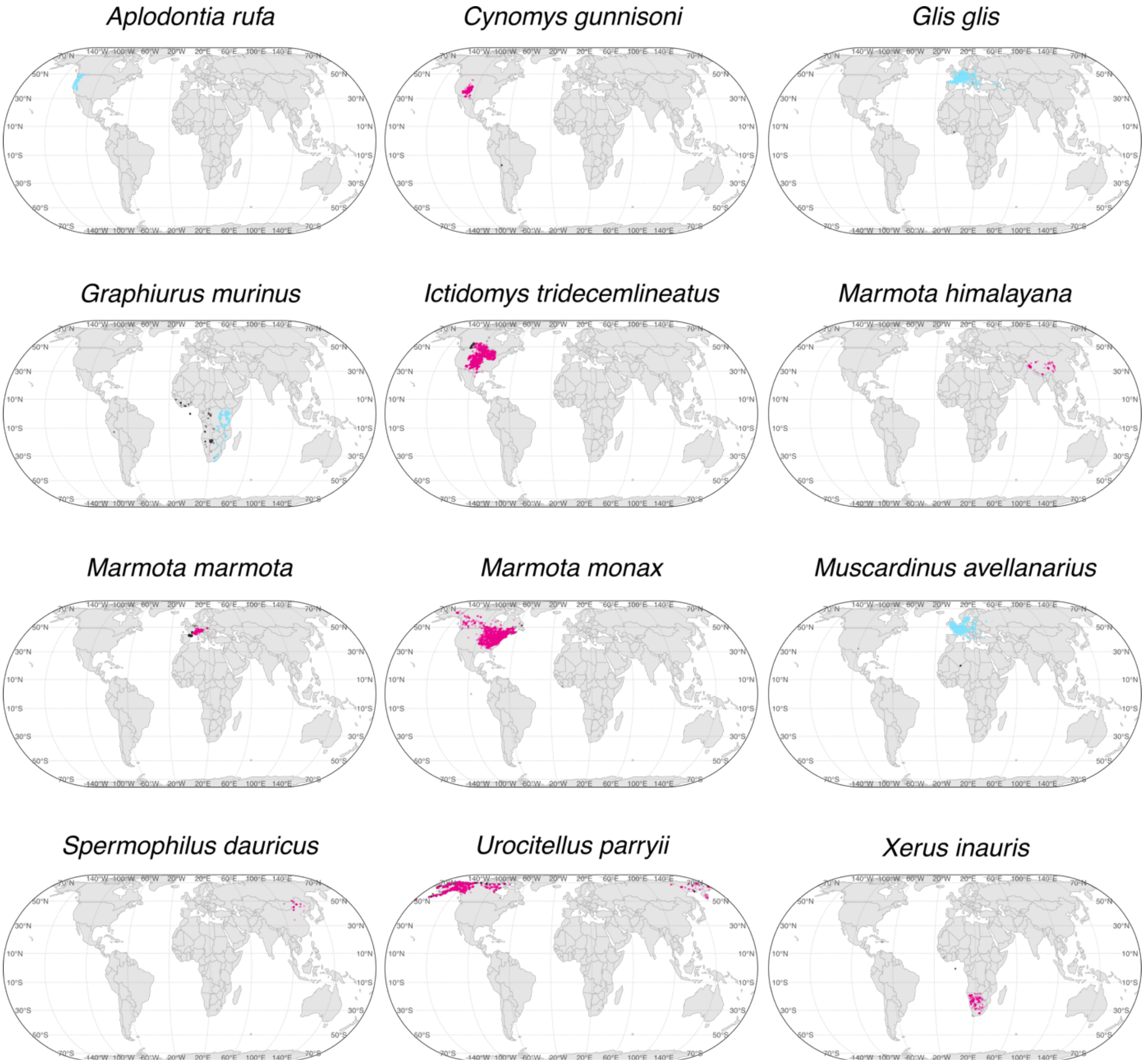

Supplemental Figure 2. Geographic distribution of individual rodent species (Sciuromorpha). Geolocation data of the indicated rodent species were extracted from the Global Biodiversity Information Facility (GBIF) and were used in order to plot their geographic distribution. Observation data was matched according to the natural distribution of each species [1, 2]. Furthermore, observations were excluded which were i) assigned zero, zero latitude and longitude (i.e. only a vague region is provided in the dataset with 0,0 coordinates given as default which maps into the Atlantic Ocean); ii) any positions that map onto water bodies (seas / oceans and large lakes observable on global maps); and iii) Any observations whose aridity values are zero as the mixed effects model requires aridity > 0 due to gamma function. All observations that were excluded from the analysis are shown in the individual maps in dark grey. Colours represent observations of species with (light blue) or without (magenta) functional *SCNN1D*. Please note that *X. inauris* is now assigend to the subgenus *Geosciurus inauris*.

1. Wilson, D.E., Lacher, T.E. Jr., Mittermeier, R.A. (**2016**): Handbook of the Mammals of the World. Volume 6: Lagomorpha and Rodents I. Barcelona: Lynx Edition.
2. Wilson, D.E., Lacher, T.E. Jr., Mittermeier, R.A. (**2017**): Handbook of the Mammals of the World. Volume 7: Rodents II. Barcelona: Lynx Edition.

Supplemental Figure 3

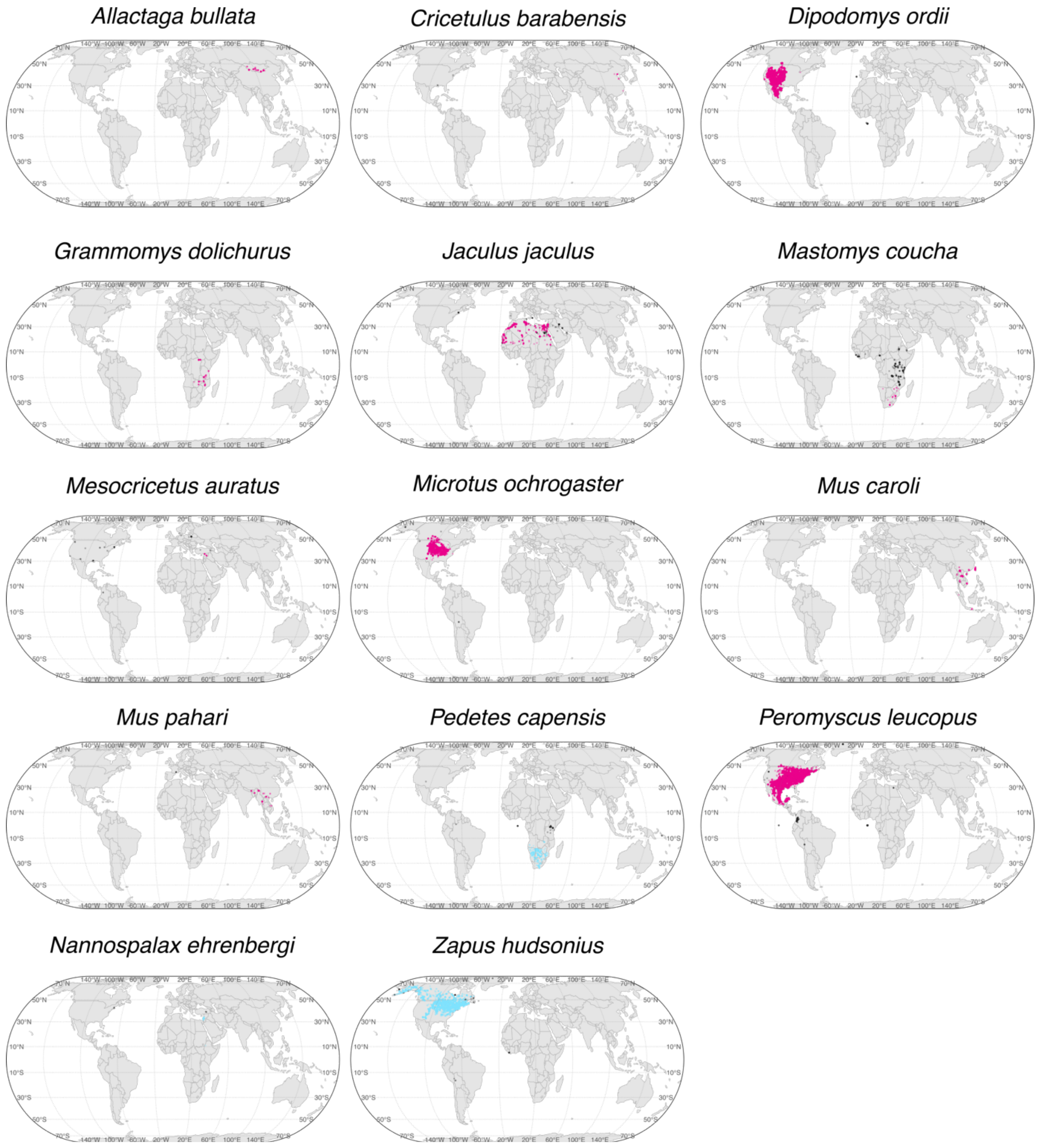

Supplemental Figure 3. Geographic distribution of individual rodent species (Supramyomorpha). Geolocation data of the indicated rodent species were extracted from the Global Biodiversity Information Facility (GBIF) and were used in order to plot their geographic distribution. Observation data was matched according to the natural distribution of each species [1, 2]. Furthermore, observations were excluded which were i) assigned zero, zero latitude and longitude (i.e. only a vague region is provided in the dataset with 0,0 coordinates given as default which maps into the Atlantic Ocean); ii) any positions that map onto water bodies (seas / oceans and large lakes observable on global maps); and iii) Any observations whose aridity values are zero as the mixed effects model requires aridity > 0 due to gamma function. All observations that were excluded from the analysis are shown in the individual maps in dark grey. Colours represent observations of species with (light blue) or without (magenta) functional *SCNN1D*. Please note that *A. bullata* is now assigend to the subgenus *Orientallactaga bullata*.

1. Wilson, D.E., Lacher, T.E. Jr., Mittermeier, R.A. (**2016**): Handbook of the Mammals of the World. Volume 6: Lagomorpha and Rodents I. Barcelona: Lynx Edition.
2. Wilson, D.E., Lacher, T.E. Jr., Mittermeier, R.A. (**2017**): Handbook of the Mammals of the World. Volume 7: Rodents II. Barcelona: Lynx Edition.

Supplemental Figure 4

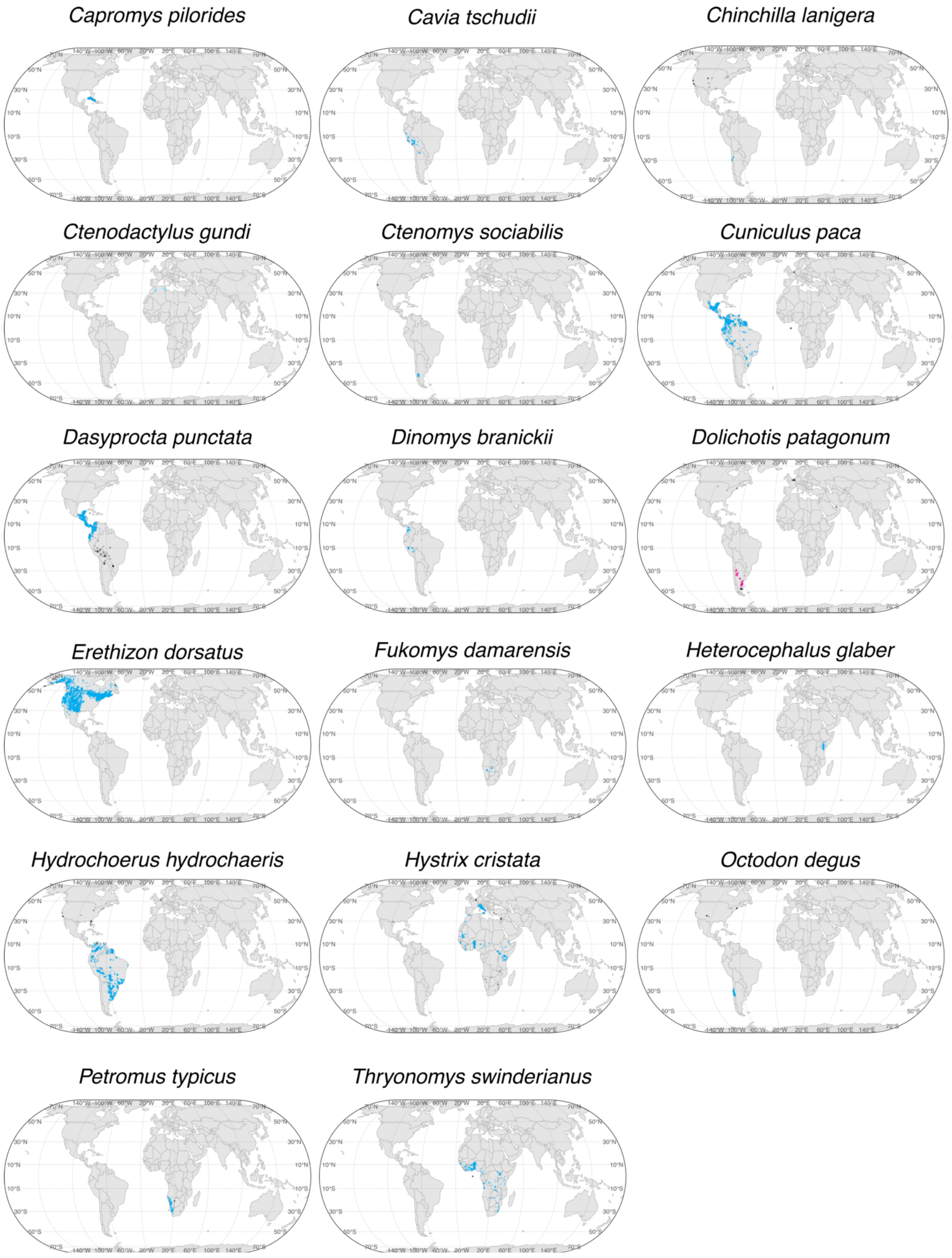

Supplemental Figure 4. Geographic distribution of individual rodent species (Hystricomorpha). Geolocation data of the indicated rodent species were extracted from the Global Biodiversity Information Facility (GBIF) and were used in order to plot their geographic distribution. Observation data was matched according to the natural distribution of each species [1, 2]. Furthermore, observations were excluded which were i) assigned zero, zero latitude and longitude (i.e. only a vague

region is provided in the dataset with 0,0 coordinates given as default which maps into the Atlantic Ocean); ii) any positions that map onto water bodies (seas / oceans and large lakes observable on global maps); and iii) Any observations whose aridity values are zero as the mixed effects model requires aridity > 0 due to gamma function. All observations that were excluded from the analysis are shown in the individual maps in dark grey. Colours represent observations of species with (blue) or without (magenta) functional *SCNN1D*. Please note that *F. damarensis* was previously named *Cryptomys damarensis* [3].

1. Wilson, D.E., Lacher, T.E. Jr., Mittermeier, R.A. (**2016**): Handbook of the Mammals of the World. Volume 6: Lagomorpha and Rodents I. Barcelona: Lynx Edition.
2. Wilson, D.E., Lacher, T.E. Jr., Mittermeier, R.A. (**2017**): Handbook of the Mammals of the World. Volume 7: Rodents II. Barcelona: Lynx Edition.
3. Kock, D., Ingram, C. M., Frabotta, L. J., Honeycutt, R. L., & Burda, H. (**2006**): On the nomenclature of Bathyergidae and Fukomys n. gen. (Mammalia: Rodentia). Zootaxa. https://doi.org/10.11646/zootaxa.1142.1.4

Supplemental Figure 5

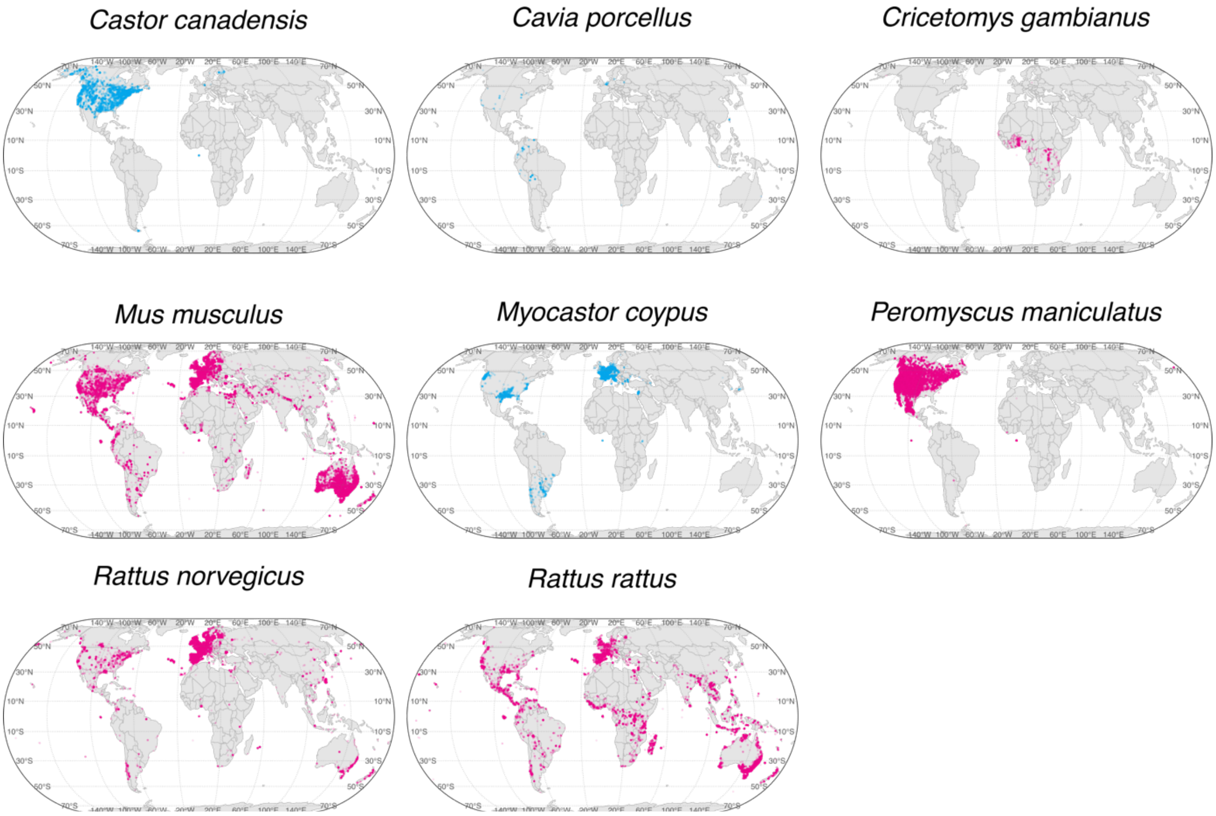

Supplemental Figure 5. Geographic distribution of invasive rodent species that were excluded from the geographic distribution analysis. Geolocation data of the indicated rodent species were extracted from the Global Biodiversity Information Facility (GBIF) and were used in order to plot their geographic distribution. Colours represent observations of species with (blue) or without (magenta) functional *SCNN1D*.
